# Safety, efficacy, and impact on gut microbial ecology of a *Bifidobacterium longum* subspecies *infantis* LMG11588 supplementation in healthy term infants: a randomized, double-blind, controlled trial in the Philippines

**DOI:** 10.3389/fnut.2023.1319873

**Published:** 2023-12-14

**Authors:** Maria Rosario Z. Capeding, Loudhie Cyd M. Phee, Chang Ming, Mario Noti, Karine Vidal, Gilles Le Carrou, A. Frézal, Janne Marie Moll, Josef Korbinian Vogt, Pernille Neve Myers, Bjørn Henrik Nielsen, Claire L. Boulangé, Tinu Mary Samuel, Bernard Berger, Colin Ivano Cercamondi

**Affiliations:** ^1^Asian Hospital and Medical Center, Muntinlupa City, Philippines; ^2^Biostatistics & Data, Nestlé Research, Lausanne, Switzerland; ^3^Nestlé Institute of Health Sciences, Nestlé Research, Société des Produits Nestlé S.A., Lausanne, Switzerland; ^4^Clinical Microbiomics, Copenhagen, Denmark; ^5^Nestlé Product Technology Center – Nutrition, Société des Produits Nestlé S.A., Vevey, Switzerland

**Keywords:** *B. infantis* LMG11588, infant growth, safety, *Bifidobacterium*-rich microbiota, autochthonous strains

## Abstract

**Introduction:**

*Bifidobacterium longum* subspecies infantis (*B. infantis*) may play a key role in infant gut development. This trial evaluated safety, tolerability, and efficacy of *B. infantis* LMG11588 supplementation.

**Methods:**

This randomized, placebo-controlled, double-blind study conducted in the Philippines included healthy breastfed and/or formula-fed infants (14–21 days old) randomized for 8 weeks to a control group (CG; *n* = 77), or any of two *B. infantis* experimental groups (EGs): low (Lo-EG; 1*10^8^ CFU/day; *n* = 75) or high dose (Hi-EG; 1.8*10^10^ CFU/day; *n* = 76). Primary endpoint was weight gain; secondary endpoints included stooling patterns, gastrointestinal symptoms, adverse events, fecal microbiome, biomarkers, pH, and organic acids.

**Results:**

Non-inferiority in weight gain was demonstrated for Hi-EG and Lo-EG vs. CG. Overall, probiotic supplementation promoted mushy-soft stools, fewer regurgitation episodes, and increased fecal acetate production, which was more pronounced in the exclusively breastfed infants (EBF) and positively correlated with *B. infantis* abundance. In EBF, fecal pro-inflammatory cytokines (IL-1 beta, IL-8) were reduced. Strain-level metagenomic analysis allowed attributing the increased abundance of *B. infantis* in EGs versus CG, to LMG11588 probiotic colonization. Colonization by autochthonous *B. infantis* strains was similar between groups.

**Discussion:**

*B. infantis* LMG11588 supplementation was associated with normal infant growth, was safe and well-tolerated and promoted a *Bifidobacterium*-rich microbiota driven by *B. infantis* LMG11588 colonization without disturbing the natural dispersal of autochthonous *B. infantis* strains. In EBF, supplementation stimulated microbial metabolic activity and beneficially modulated enteric inflammation.

## Introduction

Bifidobacteria begin to flourish and dominate the infant gut microbiota after the initial facultative anaerobic colonizers have depleted oxygen in the gut (1). Depending on gestational age (2), delivery mode (3), feeding type (breastmilk or formula) (4), and medication use (5), the highest bifidobacteria abundances are found in healthy, full-term and breastfed infants (6). *Bifidobacterium* (*B.*) species, such as *B. breve, B. bifidum, B. longum* subspecies *longum*, and *B. longum* subspecies *infantis (B. infantis)* are highly specialized in human milk oligosaccharides (HMOs) utilization (7) which may give these species an ecological niche advantage in the breastfed infant gut and explains why these species are generally more abundant in breastfed compared to formula-fed infants. Bifidobacteria inhibit the growth of pathogenic bacteria through the production of lactate and acetate resulting in a decrease of intraluminal pH (8). In particular, *B. infantis* has been associated with increased short chain fatty acid (SCFA) production resulting in an acidified gut environment (9) and possibly provide additional benefits, such as serving as energy source for neighboring colonocytes, improving gut barrier integrity, and immune system stimulation (10, 11).

Recent studies have shown a decrease of overall bifidobacteria in breastfed infants, and specifically *B. infantis*, as well as an increased abundance of bacteria taxa found associated with dysbiosis in infants, particularly in the developed world (12–14). A balanced gut microbiota rich in HMO-utilizing bacteria is considered critical for immune system maturation in infants, therefore, *B. infantis* supplementation is a promising approach to support such microbiota. In studies with small sample size, supplementation of *B. infantis* strain ATCC 15697 had limited effects on the microbiota of premature and diseased infants (15–17). In larger studies in healthy infants, supplementation with other *B. infantis* strains, EVC001 and R0033, was safe, well-tolerated and conferred beneficial effects on intestinal microbiota, metabolic and inflammatory profiles (12, 13, 18–20).

The *B. infantis* strain LMG11588 has not been previously evaluated in a clinical setting. Therefore, this randomized, double-blind, placebo-controlled trial aimed to evaluate the safety and efficacy of the *B. infantis* strain LMG11588 in early infancy. We tested two different daily dosages, hypothesizing that supplementation up to 1.8*10^10^ CFU *B. infantis* LMG11588 per day would be safe and well-tolerated and show beneficial effects on gut microbiota and gut health.

## Methods

### Design

This study was conducted in City of Muntinlupa, Philippines and included three arms: a placebo control group (CG), an experimental group (EG) receiving a high daily dose of *B. infantis* (Hi-EG), and one receiving a low daily dose of *B. infantis* (Lo-EG). Infants aged 14–21 days were randomized to one of the groups using Medidata Balance with the dynamic allocation algorithm within strata of sex (female/male), mode of delivery (C-section/vaginal) and feeding regimen (breastfeeding, formula-feeding, mixed feeding). Study investigators were blinded to group assignment and outcome assessment. At baseline, parent (s)/legally authorized representative (LAR) provided written informed consent. The study was approved by the Institutional Review Board at Asian Hospital and Medical Centre Research Ethics Committee and registered on ClinicalTrials.gov (NCT04765852).

The *B. infantis* LMG11588 strain was selected for this clinical trial based on its lack of antibiotic resistance and its capacity to use the abundant fucosylated human milk oligosaccharides (21). The Hi-EG supplement contained 1.8*10^10^ CFU *B. infantis* while the Lo-EG supplement contained 1.0*10^8^ CFU *B. infantis*, both with maltodextrin as excipient. The placebo supplement contained only the maltodextrin excipient. The placebo and *B. infantis* supplements were provided in powder-form in stick packs requiring reconstitution with approximately 10 mL of breastmilk or formula prior to feeding. All supplements were consumed orally once daily using a specific infant feeding cup, preferably in the morning. The intervention was administered for 8 weeks, and infants were followed-up for additional 4 weeks. Infants attended study visits at baseline (V1, age 14–21 days), study day 28 ± 3 (V2, 1.5 month of age), study day 56 ± 3 (V3, 2.5 month of age) and study day 84 ± 3 (V4, 3.5 month of age).

The primary outcome was weight gain from baseline (V1) to 8 weeks of intervention (V3). Secondary outcomes included additional anthropometrics (weight, length, head circumference, and corresponding *z*-scores), gastrointestinal (GI) symptoms and associated behaviors, stool characteristics, illness symptoms, adverse events (AE), fecal microbiome, fecal metabolic profile, and fecal markers of gut and immune health.

### Participants

Infants were enrolled at 14–21 days of age if they met the following inclusion criteria: full-term (37–42 weeks of gestation); birth weight ≥ 2,500 g and ≤4,500 g; exclusively breastfed, exclusively formula-fed, or mixed fed whose parent (s)/LAR did not intend to change the feeding regimen until study end; formula-fed infants could tolerate a standard cow’s milk infant formula not containing any probiotics at time of enrolment. See Supplementary material for exclusion criteria.

### Safety and tolerance outcomes

Trained study personnel obtained anthropometric measures at V1, V2, and V3, as described in Supplementary material. A one-day GI Symptom and Behavior diary capturing frequency of vomiting/spitting-up, flatulence and crying/fussiness, sleep duration as well as stooling frequency, difficulty in passing stool, stool consistency was retrospectively completed by parents at V1, and then a prospective three-day GI Symptom and Behavior diary capturing the same parameters was completed by the parent (s)/LAR just prior to V2 and V3 (see details in Supplementary material). Overall GI tolerance was measured at V1, V2, and V3 via the validated Infant Gastrointestinal Symptom Questionnaire-13 (IGSQ) which includes 13 questions covering 5 domains (stooling, vomiting/spit-up, crying, fussiness, and flatulence) (22).

Predefined illness symptoms (fever, respiratory tract symptoms, gastrointestinal symptoms, and ear symptoms) were recorded at V1, V2, and V3 by the parents/LAR on daily basis using a calendar-based infant illness diary. Standard AE reporting was done at each study visit and all parents/LAR-reported and physician-confirmed AEs were categorized using the Medical Dictionary for Regulatory Activities (MedDRA).

### Analysis of fecal pH, organic acids, gut and immune health biomarkers

Fecal pH and organic acids were assessed at V1, V2, and V3 using pH-indicator paper (Merck, Darmstadt, Germany) and validated LCMS, respectively (23). ELISA kits were used to analyze fecal biomarkers at V1, V2, and V3 including secretory immunoglobulin A (sIgA), calprotectin (Immundiagnostik AG, Bensheim, Germany) and alpha-1-antitrypsin (AAT) (BioVendor – Laboratorni medicina a.s., Brno, Czech Republic). Fecal cytokines were quantified as previously published (24), diluted 1:2 in Meso Scale Discovery diluent (MSD; Rockville, MD, United States), using V-Plex Plus kits and U-plex according to manufacturer’s instructions, and a QuickPlex SQ 120 Imager (MSD; Rockville, MD, United States). Total protein content in fecal extracts was quantified using Pierce BCA protein assay kit (Thermo Scientific) and used for cytokine level normalization.

### Microbiome analysis and ecological measures

DNA extraction, sequencing, data preprocessing, read mapping, taxonomical profiling using metagenomic species (MGS) signature genes, taxonomical annotation, microbiota diversity calculation, and pathogenic species detection was performed as previously described (25) except for the use of updated gene and MGS catalogs containing 20,992,485 genes and 1,472 MGS, respectively (see details in Supplementary material).

### Fecal community types

Fecal community type (FCT) clustering was performed using Dirichlet Multinomial Mixture modelling (26) on relative genus-level abundance data, aggregating unclassified taxa according to their best taxonomic level. Models were run fitting from 1 to 15 Dirichlet components (i.e., clusters) over 10 iterations. Models were evaluated based on Laplace approximation (Laplace), Akaike information criterion (AIC), and Bayesian information criterion (BIC). FCTs were ordered according to chronological prevalence.

### Strain profiling of *B. infantis*

A phylogenetic tree for *B. infantis* was built based on single nucleotide variant (SNV) information in sample reads as well as simulated error-free reads from reference genomes of *B. infantis* LMG11588, 16 publicly available *B. infantis* genomic sequences (27), and *B. longum* subspecies *longum* JCM 1217. SNVs at each position of 100 *B. infantis* MGS signature genes optimized for accurate abundance profiling were identified using BCFtools multiallelic-caller (v.1.11) (28, 29) retaining all alternative alleles with allele frequency ≥90% in samples with at least 250 reads mapping to at least 10 signature genes. Genes were excluded if more than 20% of its positions had allele frequencies <90%. Sample-specific inferred gene sequences were concatenated after trimming uncalled positions and used as input for one multiple sequence alignment. The phylogenetic tree was inferred using IQtree2 (v. 2.1.2) (30, 31) using *B. longum* subspecies *longum* as an outgroup. ModelFinder was used to select a substitution model for each of the genes (32–44).

### Statistics

A sample size of 76 infants per group was determined based on a non-inferiority margin of 3.6 g/day, a standard deviation of 7.7, and 3% attrition. The non-inferiority margin of 3.6 g/day was based on a prior study of infants in the Philippines (45), in which the mean (SD) for normal weight gain from birth to 4 months of age was 36 (7.7) g/day and the non-inferiority standard by the American Academy of Pediatrics (46), which established a difference of minus 10% from normal weight gain between 0 to 4 months of age as clinically relevant. Non-inferiority was concluded if the lower bound of the two-sided 95% confidence interval of the difference between the Hi-EG and control groups excluded −3.6 g/day.

The primary endpoint of weight gain was analyzed using analysis of covariance (ANCOVA) correcting for baseline weight and sex in both the full analysis set (FAS) and per-protocol set (PPS). FAS included all subjects who received at least one dose of probiotic or placebo supplement, and PPS included all compliant subjects who consumed the assigned supplement for 80% of study days. Secondary endpoints were analyzed in the FAS population. All analyses, except for microbiome, were conducted using SAS statistical software Version 9.4. Statistical significance was tested at the two-sided 5% level. *P*-values <0.05 were considered significant (except for microbiota analysis; see below) and Benjamini-Hochberg correction was applied for any multiple testing. Anthropometric were analyzed using ANCOVA, correcting for each respective baseline value and sex. The IGSQ-13 index score, mean stool frequency, difficulty in passing stool, and mean stool consistency were analyzed using ANCOVA correcting for baseline value. Group comparisons of GI symptoms and GI related behaviors, and AEs of interest were made using the Fisher–Halton test and the Benjamini–Hochberg correction for multiple testing. The relative risk of each illness symptom was calculated for Hi-EG and Lo-EG versus CG as reference.

For pH, organic acids, biomarkers and cytokines, differences between study groups were examined using ANCOVA correcting for baseline value. When less than 10% of values were below the lower limit of detection (LLD), the values below LLD were replaced with the LLD itself. When more than 50% of values for a given measure were below the limit of detection (LOD), ANCOVA modeling was not performed.

All statistical microbiota analyses were performed using R v. 4.2.1 (47). Unless otherwise stated, PERMANOVA tests were performed using the adonis2 function from the vegan R package with 1,000 permutations and by = “margin,” thus assessing the marginal effects of the terms (i.e., each marginal term analyzed in a model with all other variables). Association between biomarkers and microbiome features were investigated using Kendall Rank Correlation as implemented in the “Kendall” R package. Benjamini-Hochberg correction was used to correct for multiple testing, and *p*-values <0.05 and adjusted *p*-values <0.1 were considered significant.

## Results

**Figure 1:**
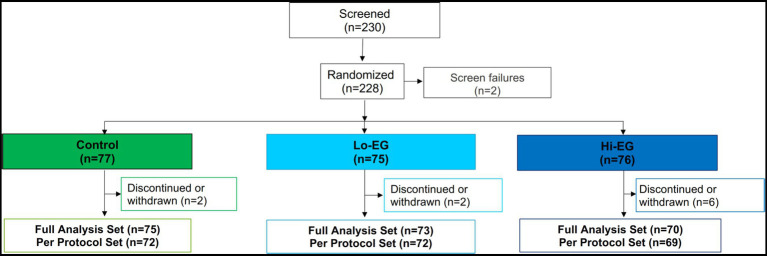
CONSORT diagram of subject disposition. Lo-EG, experimental group receiving low daily dose of *B. infantis* (1.0*10^8^ CFU); Hi-EG, experimental group receiving high daily dose of *B. infantis* (1.8*10^10^ CFU).

There were 228 infants enrolled in the study (CONSORT diagram of subject disposition, Figure 1). Baseline characteristics for the study participants in FAS were comparable between groups (Table 1). Most of the infants were born vaginally (85%), were exclusively breastfed (EBF, 58%), and only 16% were exclusively formula-fed.

**Table 1:** Baseline characteristics of study participants, FAS.

	CG (*n* = 75)	Lo-EG (*n* = 73)	Hi-EG (*n* = 70)
Age at baseline, days^a^	17.0 ± 1.9	17.3 ± 1.9	16.6 ± 1.9
Gestational age, weeks	38.9 ± 1.0	38.8 ± 0.9	38.7 ± 1.1
Type of delivery, % vaginal	84.0%	84.9%	85.7%
Infant sex, % male	54.7%	53.4%	51.4%
Birth weight, kg	3.0 ± 0.3	3.1 ± 0.4	3.0 ± 0.3
Weight at baseline, kg	3.5 ± 0.4	3.5 ± 0.4	3.4 ± 0.4
Length at baseline, cm	51.6 ± 1.7	51.6 ± 1.6	51.3 ± 1.8
Head circumference at baseline, cm	34.7 ± 1.1	34.6 ± 1.3	34.3 ± 1.2
BMI at baseline, kg/m^2^	13.0 ± 1.0	13.2 ± 1.1	13.0 ± 1.1
Maternal age, years	28.6 ± 6.0	27.9 ± 6.0	27.5 ± 6.1
Maternal education (Associate degree or college degree), %	29.3%	23.3%	28.6%
Any formula-feeding (exclusively formula-fed and mix-fed), %	44%	42%	41%
Exclusively formula-fed, %	16%	18%	14%
Mixed-fed, %	28%	24%	27%
Exclusively breastfed, %	56%	58%	59%

FAS, full analysis set; CG, control group (placebo supplement); Lo-EG, low-dose *B. infantis*, 1.0 × 10^8^ CFU; Hi-EG, high-dose *B. infantis*, 1.8 × 10^10^ CFU.

a Data presented are mean ± SD unless otherwise specified.

### Primary growth outcome and additional anthropometrics

Weight gain in g/day from enrollment through 8 weeks for FAS averaged 35.0 (95% CI: 33.3, 36.8) in CG, 35.6 (95% CI: 33.8, 37.4) in Lo-EG, and 36.5 (95% CI: 34.6, 38.3) in Hi-EG. Non-inferiority in daily weight gain was demonstrated for Low-EG and Hi-EG compared to CG in both FAS and PPS, with the lower bound of the 95% confidence interval above the predefined non-inferiority margin of −3.6 g/day (*p* < 0.05 for both) (Table 2). In FAS, gains in body weight, length, and head circumference from enrollment to the end of the study period were comparable among the three groups and increased over time. Mean weight-for-age, length-for-age, weight-for-length and head circumference-for-age, *z*-scores demonstrated comparable growth among all groups (Supplementary Figure S1).

**Table 2:** Difference in weight gain (g/day) from enrollment to study completion at 2.5 months of age (primary outcome), FAS and PPS.

Population	Group	Adjusted^a^ mean weight gain in g/day (95% CI)	Group comparisons		
			Comparison	Estimate of mean difference (95% CI)^b^	Non-inferiority met^c^ (value of *p*)
FAS	CG	35.0 (33.3, 36.8)	–	–	–
	Lo-EG	35.6 (33.8, 37.4)	Lo-EG vs. CG	0.52 (−2.33, 3.37)	Yes (0.0004)
	Hi-EG	36.5 (34.6, 38.3)	Hi-EG vs. CG	1.42 (−1.47, 4.30)	Yes (0.0008)
PPS	CG	34.8 (33.0, 36.6)			
	Lo-EG	35.7 (33.8, 37.5)	Lo-EG vs. CG	0.83 (−2.07, 3.73)	Yes (0.0005)
	Hi-EG	36.5 (34.6, 38.3)	Hi-EG vs. CG	1.66 (−1.27, 4.59)	Yes (0.0004)

FAS, full analysis set; PPS, per protocol set; CG, control group (placebo supplement); Lo-EG, low-dose *B. infantis*, 1.0 × 10^8^ CFU; Hi-EG, high-dose *B. infantis*, 1.8 × 10^10^; CI, confidence interval.

a From an ANCOVA model correcting for baseline weight and sex.

b CIs are adjusted by Tukey correction.

c Non-inferiority of experimental vs. control group on weight gain from enrollment to age 2.5 months accepted if the lower bound of the two-sided 95% CI on the model-based group difference was above the non-inferiority margin of −3.6 g/day.

### GI tolerance and associated behaviors

Mean IGSQ-13 scores were below the threshold of 23 for GI distress at V2 and V3 and were comparable among the groups (Supplementary Table S1). Additional GI symptoms, behaviors, and stooling characteristics are summarized in Supplementary Table S2. Frequency of regurgitation/vomiting differed between Hi-EG and CG at Visit 2 with infants in Hi-EG having a lower frequency of vomiting compared to CG (value of *p* = 0.031). At Visit 3, frequency of vomiting was lower in both Hi-EG and Lo-EG compared with CG (value of *p* = 0.044 and 0.006, respectively). Flatulence was consistently rare across all three groups, with an overwhelming majority of infant caregivers reporting zero or one occasion. Crying and fussiness was predominantly absent.

Stool frequency and consistency scores were similar between groups at each timepoint. By consistency category, most stools were “soft” at both V2 and V3 for all three groups, but significant differences were observed at V2 for Hi-EG compared to both CG (*p* < 0.0001) and Lo-EG (*p* < 0.0001), driven by more stools reported as watery and runny in Hi-EG. Lo-EG also differed from CG at V2 (value of *p* = 0.021) with more stools reported as soft and fewer stools reported as formed. There were very few occurrences of difficulty in passing stool throughout the study.

### Adverse events and illness symptoms

Overall, an AE was reported in 37–43% of the infants across the three study groups (with upper respiratory tract infection being most common at 22.5% overall), and one subject in CG experienced a serious adverse event (SAE) (Supplementary Table S3). The severity of AEs was predominantly mild (95%). There were no group differences for AEs of interest and the AE severity or SAEs.

Symptoms reported by parents/LAR in the infant illness diary (Supplementary Table S4) were not different for fever or gastrointestinal symptoms, while the frequency of respiratory tract symptoms trended towards a lower frequency in Hi-EG and Lo-EG compared to CG (value of *p* = 0.085). The unadjusted relative risk (RR) for respiratory tract symptoms for Lo-EG versus CG was significantly reduced (RR = 0.42, 95% CI 0.19, 0.96, value of *p* = 0.044).

### Fecal pH, organic acids, gut and immune health biomarkers

At V2, but not V3, pH tended to be lower in Hi-EG compared to CG and Lo-EG (unadjusted value of *p* = 0.051 and 0.027, respectively) (Supplementary Table S5A). In the subset of EBF infants similar observations were done but only for Hi-EG compared to CG (unadjusted value of *p* = 0.037) (Supplementary Table S5B). At V2, acetate was higher in Hi-EG compared to CG (adjusted value of *p* = 0.0023), as observed in EBF infants (adjusted value of *p* = 0.059). At V3, the difference of acetate concentration was attenuated but still showed a trend in EBF infants (adjusted value of *p* = 0.066). At V3 but not V2, acetate concentration also showed a trend to be higher in Lo-EG versus CG in EBF infants (value of *p* = 0.066). There were no consistent differences between groups for butyrate, isobutyrate, isovalerate, propionate, or valerate at V2 or V3 overall or in EBF (Supplementary Tables S5A,B).

Fecal biomarkers of immune health (sIgA), intestinal inflammation (calprotectin) and gut barrier (AAT) were largely comparable among groups (Supplementary Table S6). However, AAT was significantly higher in Hi-EG compared to CG at V2 (adjusted value of *p* = 0.042). In EBF, there was some indication of higher AAT in Hi-EG and Lo-EG compared to CG at V2 (adjusted value of *p* = 0.087 and 0.10, respectively).

For several fecal cytokines measurements, more than 50% were below the LOD, so no statistical testing was performed (Supplementary Table S7). In the overall population at either visit, there were no significant group differences for most cytokines tested (IFN-γ, IL-1β, IL-8, IL-23, and IL-27) (Supplementary Table S8). IL-1β levels were lower in Hi-EG versus CG at V2 (value of *p* = 0.031), and lower in EBF Hi-EG and EBF Lo-EG versus EBF CG at both timepoints (V2: adjusted value of *p* = 0.014 and 0.0034, respectively; V3 adjusted value of *p* = 0.015 and 0.035, respectively). In EBF, IL-8 at V3 was lower in Lo-EG vs. CG (adjusted value of *p* = 0.026) and showed a trend for Hi-EG vs. CG (adjusted value of *p* = 0.061).

### Gut microbiome inter-group comparisons

**Figure 2:**
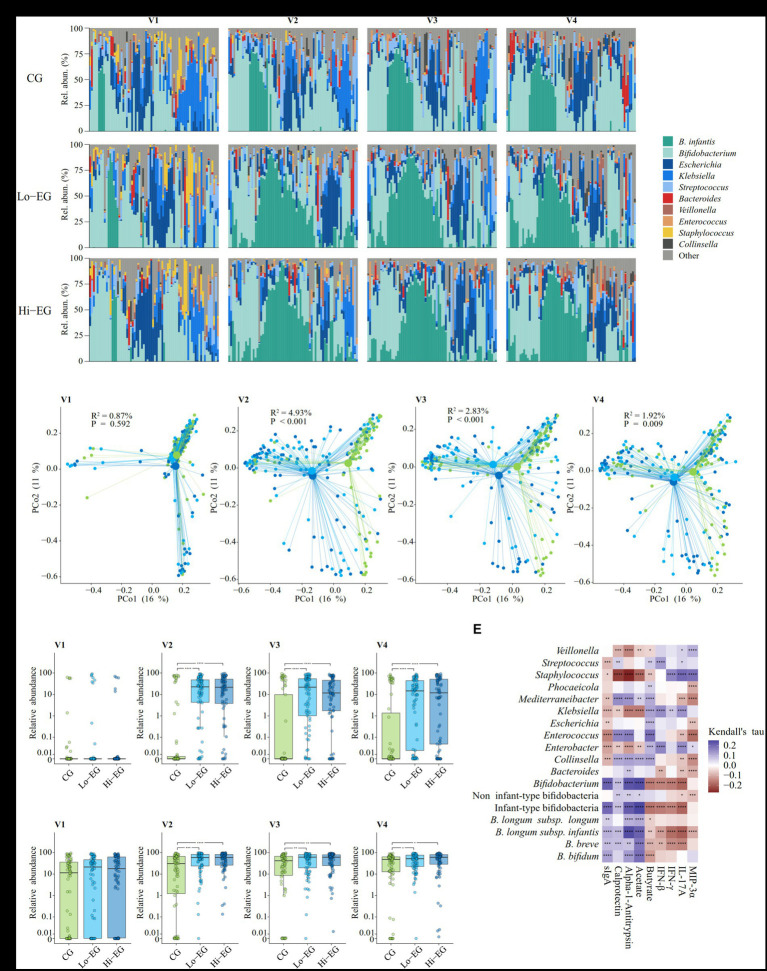
Gut microbiome composition, diversity analyses, and association with fecal biomarkers and cytokines (*n* = 865 samples, 218 infants). CG, control group (placebo supplement); Lo-EG, low-dose *B. infantis*, 1.0 × 10^8^ CFU; Hi-EG, high-dose *B. infantis*, 1.8 × 10^10^ CFU. **(A)** Overview of abundance for *B. infantis* and dominant genera across visits and intervention groups for all samples. **(B)** PCoA ordination plots of Bray–Curtis dissimilarity stratified by visit. Ordinations were calculated for all samples, making coordinates comparable between visits. Filled circles designate individual samples and lines connect them to the centroid of their respective group (large circles). Text inserts refer to variance explained and *p*-values from PERMANOVA group comparisons performed for each visit. **(C)** Relative abundance (%) of *B. infantis* among visits and groups. Box edges denote the upper and lower quartiles, the center line denotes the median, and the whiskers extend to the most extreme datapoints within 1.5 * IQR from the upper and lower quartiles, respectively. Abundances are shown on a pseudo-log scale with the linear segment ranging from 0% to 0.01%. *: *p* < 0.05, **: *p* < 0.01, ***: *p* < 0.001, and ****: *p* < 0.0001. **(D)** Relative abundance (%) of infant-type bifidobacteria across visits and groups. Box edges denote the upper and lower quartiles, the center line denotes the median, and the whiskers extend to the most extreme datapoints within 1.5 * IQR from the upper and lower quartiles, respectively. Abundances are shown on a pseudo-log scale with the linear segment ranging from 0% to 0.01%. *: *p* < 0.05, **: *p* < 0.01, ***: *p* < 0.001, ****: *p* < 0.0001. **(E)** Correlation between microbiota taxon abundances and fecal biomarkers and cytokines across all visits. Genera with an average abundance of at least 1% and Bifidobacterium taxa of interest were included in the analysis. *p*-values were adjusted using the Benjamini–Hochberg method; *: *p* < 0.10, **: *p* < 0.05, ***: *p* < 0.01, ****: *p* < 0.001.

The gut microbiome composition was investigated by shotgun metagenomics on the stool samples collected at V1 (baseline, before supplementation), V2, V3, and V4 (Figure 2A). The within-samples diversity indexes were comparable among the groups at most time points (Supplementary Table S9), except lower genus-level diversities in both Lo-EG and Hi-EG compared to CG at V2 (Supplementary Figure S2A). The overall taxonomic composition dissimilarity between groups (β-diversity) was consistently and significantly different between CG and both EGs at all timepoints except V1, but never between Lo-EG and Hi-EG (Figure 2B and Supplementary Table S10). We did not find the same differences when using overall phylogenetic distances between groups, except at V2 between CG and both EGs (Supplementary Table S10). This observation is in accordance with the results of the univariate analyses showing *B. infantis* as the only taxon consistently significantly higher in both EGs compared to CG at most post-baseline visits (Figure 2C), except at V2 where phylogenetically distantly related taxa were also modulated: *Klebsiella* (genus) and *Lachnospiraceae* (family) were increased in CG compared to EGs, while *Mediterraneibacter* (genus) was lower in Lo-EG than in Hi-EG and CG (Supplementary Table S11). At V2 and in both EGs compared to CG, *Bifidobacterium* (genus) was more abundant. At V4, besides *B. infantis*, only a *Lachnospiraceae* sp. MGS with the potential to degrade fucose and mucin and to produce acetate was significantly more abundant in CG than in EGs. The consistent expansion of *B. infantis* in the intervention group resulted in a higher abundance of infant-type bifidobacteria (*B. longum*, *B. breve*, *B. bifidum*, and *B. scardovii*, as previously defined (7)) at V2, V3, and V4 (Figure 2D and Supplementary Table S11). Noteworthy, we identify for the first time in the Philippines the transitional *B. longum* clade recently discovered in Bangladeshi children during weaning (48), at a very low prevalence in our study, as expected for this age range (data not shown).

The prevalence of intestinal pathogens was low, including *Clostridium perfringens* (found in two samples from 2 infants, 0.23% of samples, 4.13% of infants), EPEC/ETEC *Escherichia coli* (15 samples from 14 infants, 1.7% of samples, 6.4% of infants), and *Campylobacter* spp. (nine samples from 9 infants, 1.04% of samples, 4.13% of infants). *Clostridioides difficile*, which was overall more prevalent in this study (found in 10.4% of samples, 24.3% of infants) was numerically less prevalent in the EGs at V2 and V3 (Fisher’s exact test, value of *p* = 0.17 for V2, value of *p* = 0.18 for V3) (Supplementary Table S12).

### Associations between gut microbiota, acetate, and gut health biomarkers

Using a Procrustes analysis, we found significant associations between fecal biomarkers and microbiota composition at all visits (V1: Procrustes correlation (PrC) = 0.187, V2: PrC = 0.197, V3: PrC = 0.262, all visits with *p* < 0.001). When investigating the associations between specific taxa and fecal biomarkers, *B. infantis* abundance was positively correlated with levels of acetate, AAT, calprotectin, and sIgA, and negatively with levels of butyrate, IFN-β, IFN-γ, IL-1β, IL-8, IL-17A, and MIP-3α (Figure 2E). Although the correlations were stronger with *B. infantis*, similar observations were made with other infant-type bifidobacteria, indicating a possible shared effect on host physiology by these taxa. Other taxa including pathobionts showed opposite patterns, like *Enterobacter*, *Klebsiella*, *Staphylococcus*, and *Veillonella*. Specifically, *Staphylococcus* abundance was associated with a significant increase in the pro-inflammatory cytokines IFN-γ, IL-17A, and MIP-3α while sIgA, calprotectin, AAT and butyrate levels showed an inverse correlation. In EBF, the same associations were observed, except for sIgA where *B. infantis* was the only associated species/subspecies (Supplementary Figure S3).

### Longitudinal tracking of *B. infantis* strains

**Figure 3:**
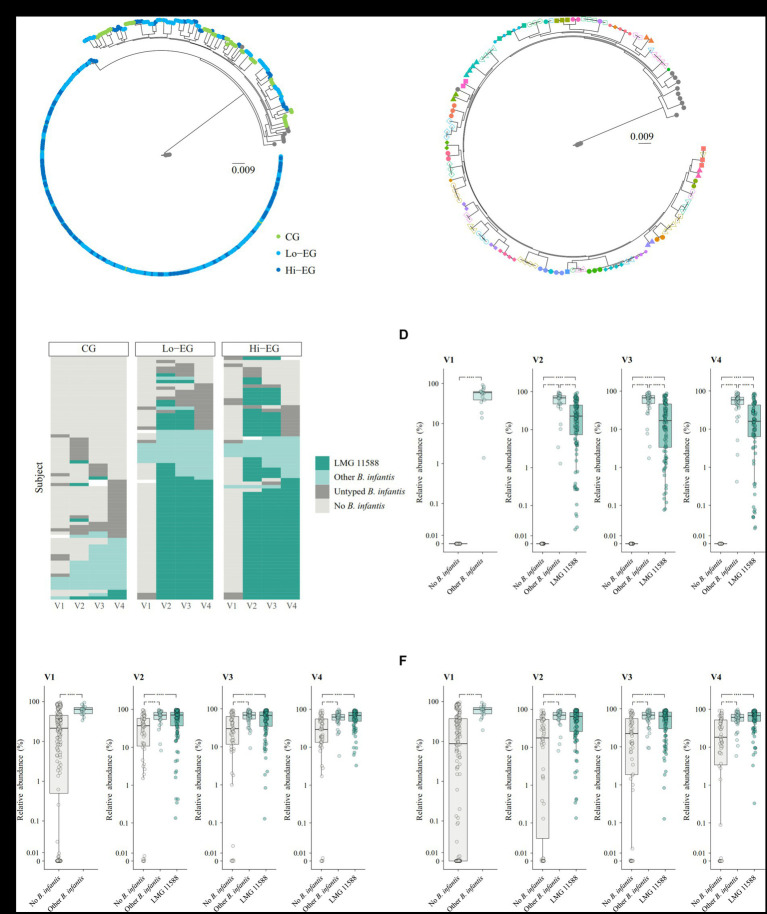
Longitudinal tracking of *B. infantis* strains. **(A)** Strain tree of *B. longum* subspecies *infantis* using the reference genome of *B. longum* subspecies *longum* JCM 1217 as root. Each tip in the tree corresponds to an infant sample (*n* = 406) or a reference genome (*n* = 16). Tip colors refer to sample groups (CG: green, Lo-EG: light blue, Hi-EG: dark blue) or reference genomes (gray). The sum of edge lengths between two tips are proportional to the number of SNV differences between the two samples. CG, control group (placebo supplement); Lo-EG, low-dose *B. infantis*, 1.0 × 10^8^ CFU; Hi-EG, high-dose *B. infantis*, 1.8 × 10^10^ CFU. **(B)** Strain tree showing infant samples (*n* = 130) and reference genomes (*n* = 15) outside the “LMG11588” clade. Tip color-shape combinations refer to the study subject ID, with reference genomes shown as gray circles. The sum of edge lengths between two tips are proportional to the number of SNV differences between the two samples. **(C)** *B. infantis* strain categorization shown for each infant (horizontal rows, *n* = 218) at the four timepoints. Missing samples are denoted with a white fill color. **(D)** Relative abundance (%) of *B. infantis* stratified according to visit and strain category (*n* = 865 samples, 218 infants). Box edges denote the upper and lower quartiles, the center line denotes the median, and the whiskers extend to the most extreme datapoints within 1.5 * IQR from the upper and lower quartiles, respectively. Abundances are shown on a pseudo-log scale with the linear segment ranging from 0% to 0.01%. *: *p* < 0.05, **: *p* < 0.01, ***: *p* < 0.001, ****: *p* < 0.0001. **(E)** Relative abundance (%) of total *Bifidobacterium* spp. stratified according to visit and strain category (*n* = 865 samples, 218 infants). Box edges denote the upper and lower quartiles, the center line denotes the median, and the whiskers extend to the most extreme datapoints within 1.5 * IQR from the upper and lower quartiles, respectively. Abundances are shown on a pseudo-log scale with the linear segment ranging from 0% to 0.01%. *: *p* < 0.05, **: *p* < 0.01, ***: *p* < 0.001, ****: *p* < 0.0001. *n* = 865 samples. **(F)** Relative abundance (%) of selected infant-type *Bifidobacterium* spp. stratified according to visit and strain category (*n* = 865 samples, 218 infants). Box edges denote the upper and lower quartiles, the center line denotes the median, and the whiskers extend to the most extreme datapoints within 1.5 * IQR from the upper and lower quartiles, respectively. Abundances are shown on a pseudo-log scale with the linear segment ranging from 0 to 0.01%. *: *p* < 0.05, **: *p* < 0.01, ***: *p* < 0.001, ****: *p* < 0.0001. *n* = 865 samples.

As supplementation with *B. infantis* LMG11588 increased the abundance of the subspecies *B. infantis*, we investigated the colonization patterns and dynamics of this probiotic strain compared to other autochtonous *B. infantis* strains, as well as their relationship with infant feeding mode and the impact on microbiota community development. Using an SNV-based strain-typing of *B. infantis*, 406 of the 507 samples with detected *B. infantis* were placed in a phylogenetic tree together with publicly available reference genomes (Figure 3A). In this tree, most samples (*n* = 277, 68.2%) fell into a clade with negligible variability that also includes the LMG11588 reference genome (henceforth named “LMG11588 clade”). The remaining samples were positioned in a clade (“Other *B. infantis*”) with higher variability (Figure 3B). The remaining 101 samples could not be unambiguously typed (“Untyped *B. infantis*”), either due to low *B. infantis* abundance (<0.16%, *n* = 95) or potential heterogeneous strains profiles (*n* = 6).

When tracking the longitudinal development of *B. infantis* strains profiles according to supplementation (Figure 3C), *B. infantis* was detected in 16% of the infants at baseline, with similar proportions between the groups (*Χ*^2^ test, value of *p* = 0.755) and none of these *B. infantis* strains placed in the LMG11588 clade. During the intervention, we identified nine instances of the probiotic strain in the control group. In all three groups, the proportion of infants with “Other *B. infantis*” increased gradually over time and reached 18.4% at visit 4 with similar “Other *B. infantis* “colonization patterns between groups (*Χ*^2^ test, *p* = 0.739). Samples from the same infant with “Other *B. infantis*” were always located in the same subclade in the phylogenetic tree (Figure 3B). In both EGs, we detected the probiotic strain in 78.7% of the infants during the intervention (V2 and/or V3) and it persisted in 70.3% of these infants 4 weeks after cessation of probiotic administration (V4). No difference in persistence of the probiotic was observed between Lo-EG and Hi-EG (*p* = 0.484). In these two groups, only one case of LMG11588 replaced an “Other *B. infantis*” strain, while in 11 infants, LMG11588 was replaced with “Other *B. infantis*” strains.

We investigated the relationship between feeding and the strains colonization pattern (Supplementary Figure S4). Differences between the three feeding groups (formula, mixed, or EBF) were only observed at V4 where exclusive breastfeeding was associated with higher proportions of colonization by any *B. infantis* strain (*Χ*^2^ test, value of *p* = 0.0196), in particular by LMG11588 (*Χ*^2^ test, value of *p* = 0.0221). During the intervention, LMG11588 colonization was observed in 76.5% of the EBF infants and the probiotic persisted in 83.9% of these infants after cessation of the supplementation. In the infants colonized with “Other *B. infantis*,” the abundance of *B. infantis* was on average higher than in those colonized with LMG11588 (Figure 3D). However, the abundances of overall bifidobacteria (Figure 3E) and infant-type bifidobacteria (Figure 3F) were comparable between infants colonized with “Other *B. infantis*” or LMG11588 and were lower in infants not colonized with *B. infantis*.

**Figure 4:**
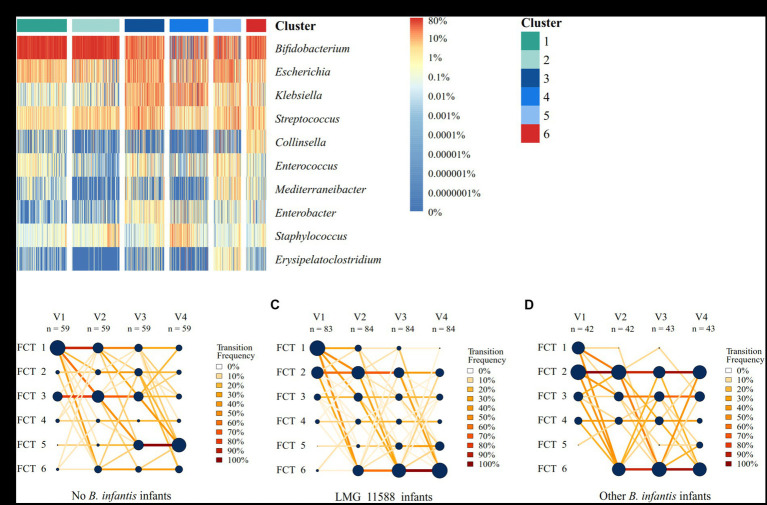
Fecal community type (FCT) analysis. **(A)** Relative abundances of the top 10 abundant genera across 865 samples (218 infants) assigned to the six different FCTs. **(B–D)** Infant transition visualization showing the progression of samples through each FCT for infants with no *B. infantis* **(B)**, with *B. infantis* LMG11588 **(C)**, and “Other” *B. infantis* strains **(D)**. Nodes represent each FCT at each visit. Node sizes represent the fraction of infants in the given FCT at the given visit (relative to the infants in the other FCTs at the same visit). Line widths represent the number of infants who transitioned from a given FCT to another FCT between two visits. Line colors represent the fraction of infants who transitioned from a given FCT to another FCT between two visits (relative to the infants who transitioned from the same FCT to the remaining FCTs at the same visit).

Finally, to test if the microbiota community development was different depending on the colonizing *B. infantis* strains at the end of the study (V4), we performed a fecal community type (FCT) analysis. Using Dirichlet Multinomial Modeling at genus-level (26), we identified six FCTs sequentially ordered based on their chronological prevalence (Figure 4A). At none of the visits, the FCT trajectories of infants colonized by LMG11588 were significantly different from the ones of infants colonized by” Other *B. infantis*” (Figures 4C,D and Supplementary Table S13), sharing FCT 2 (dominated by *Bifidobacterium*) and FCT 5 (dominated by *Escherichia* and *Streptococcus*) as dominant communities. This observation contrasts with infants not colonized by any *B. infantis*, who had trajectories significantly different from the other infants (Figure 4B and Supplementary Table S13) and showing FCT 3 (dominated by *Bifidobacterium* and *Escherichia*) as a major early FCT and FCT 5 at later visits.

## Discussion

We demonstrated that supplementation of two different doses of *B. infantis* strain LMG11588 during early infancy were safe, well-tolerated, and associated with a non-inferior daily weight gain compared to a placebo. These results are in line with studies examining other *B. infantis* strains including ATCC 15697 (15–17), EVC001 (12, 13, 20), and R0033 (19) (the latter is considered genetically identical to LMG11588). Overall incidence of AEs was similar across groups, also consistent with studies using EVC001 and R0033 strains. Interestingly, supplemented groups showed a trend towards fewer parent reported respiratory tract symptoms. Also, in both intervention groups compared to placebo, regurgitation/vomiting episodes decreased over time, similar to findings in another study of infant formula supplemented with *B. infantis* (49). Regarding infant stools, both doses showed stool characteristics similar to those observed in the study using R0033 (19), i.e., were similar compared to a placebo group and had stools generally graded as soft.

Notably, in our study these clinical observations were made at 1.5 months of age (V2) when the microbiome analyses captured transiently more pronounced changes in alpha (within samples) and beta (between groups) diversities. In our study, *B. infantis* was the only taxon significantly and consistently increased by the probiotic supplementation, except at V2, where the number of differentially abundant taxa between the EGs and the CG was higher compared to V3 and V4. Among these modulated taxa, some pathobionts were decreased, as previously observed following *B. infantis* EVC001 supplementation (13). The consistent expansion of *B. infantis* in the supplemented groups was parallel to a significantly higher abundance of bifidobacteria and more specifically the infant-type bifidobacteria (7), indicating that its increase does not occur at the expense of the abundance of other infant-associated bifidobacteria species.

In most samples with sufficient abundance of *B. infantis* (>0.16%), we could define the colonizing strains, being the probiotic strain *B. infantis* LMG11588 or autochthonous strains naturally occurring in this population of infants. Although the supplementation led to a rapid increase in the number of infants with a gut microbiota harboring *B. infantis* LMG11588 (79% of the infants), this did not occur at the expense of cases of colonization by other *B. infantis* strains, which expanded at comparable rates between the supplemented and control groups (18% of the infants at V4). In these infants showing other *B. infantis* strains, all samples from the same infant harbored the same subclade, indicating that naturally occurring *B. infantis* strains entered a stable niche that is resistant to potential perturbation by probiotic supplementation.

It is well established that HMOs play diverse and important roles in infant development starting with their prebiotic function which helps establish and maintain a balanced gut microbiota (50–53). Notably, in our study, formula-fed infants did not receive HMO-containing formulas, nor prebiotic-containing formulas (with two exceptions for the latter). Since *B. infantis* is highly specialized in utilizing HMOs (7), it was not surprising that the probiotic sustenance was supported in EBF infants at V4 (84% of persistence versus 70% in all infants). A similar colonization persistence was previously observed in breastfed infants >30 days after *B. infantis* EVC001 supplementation ceased (13). Notably, the identification of LMG11588 genomic information in the samples 1 month after cessation of supplementation (at V4) demonstrated the viability and persistence of the probiotic.

In an ecologic framework, the human gut microbiota has been proposed to be a meta-community in which individuals are linked through dispersal which shapes the microbiome assembly at local scales (54). For example, during the intervention, we detected the probiotic strain in nine samples in the control group, which may be attributed to exposure to the probiotic in the environment through horizontal transmission at social gatherings of families, infant playground, or community events. In populations with low occurrence of this horizontal transmission, the probiotic supplementation could be seen as a way to restore the dispersal process of *B. infantis* (14). Although not clearly specified in the original reference of the LMG11588 probiotic strain (55), the isolation from infant feces was performed in the United States, a country harboring a very low prevalence of *B. infantis* (14). Interestingly, our probiotic supplementation in Filipino infants did not interfere with the natural dispersal of autochthonous *B. infantis* strains. We could therefore hypothesize that, in their natural environment, the autochthonous strains out-compete the supplemented probiotic strain. Similarly, an autochthonous *B. infantis* strain Bg_2D9 was shown experimentally to have a superior fitness over the U.S. infant-derived *B. infantis* EVC001 in the context of Bangladeshi infants with severe acute malnutrition, and its competitive advantage was proposed to be brought by local complementary food (56). Since this feeding stage is not covered in our study, the nature of the competing advantage of the autochthonous strains still needs to be defined. Anyhow, it is an interesting property of the LMG11588 strain to help the establishment of a sustained *B. infantis*-rich microbiota only in infants that do not acquire it shortly after birth.

Alterations in the microbiota composition in early life influence immune system maturation and are associated with an increased risk of developing autoimmune and allergic diseases later in life (57–59). Therefore, impaired immune system development may be a consequence of gut dysbiosis and associated enteric inflammation. Interestingly, the presence of bifidobacteria strains (e.g., *B. infantis*) have been shown to reduce the risk of immune mediated disorders, likely through promoting a healthy immune system imprinting during a critical window that may impact the health trajectory of the infant (60, 61). Infant-type bifidobacteria derived metabolites such as aromatic lactic acids are known to beneficially modulate immune function and pathogen resistance in early life (7). Along the same line, the intervention group with the highest probiotic dose was characterized by a general decrease in pH and an increase of acetate production. These observations were made at V2 and were generally more pronounced and sustained in EBF infants. Increased acetate and decreased pH were previously observed in breastfed infants supplemented with *B. infantis* EVC001 (13). While we found little variation in measurable cytokines between study groups, fecal IL-1β and IL-8 levels were significantly reduced at both V2 and V3 in exclusively breastfed infants supplemented with *B. infantis* LMG11588. These results are consistent with a previous report demonstrating reduced enteric inflammation in breastfed infants supplemented with *B. infantis* EVC001 (12, 61). The observed reduction of enteric inflammation in the breastfed group and not the formula-fed groups supports the hypothesis of a synbiotic effect between HMOs and the supplemented *B. infantis* strains highly specialized in the utilization of HMOs (7, 62). Importantly, acetate- and cytokine-levels correlated with the abundance of *B. infantis*, suggesting a dose dependent effect of the probiotic *B. infantis* LMG11588 on these biomarkers of gut and immune health. However, the potential synbiotic effect to regulate enteric inflammation in early life warrants further clinical investigation.

Our study is the first demonstration of the safety and efficacy of two doses of the *B. infantis* LMG11588 strain. A main strength of the study is that it overcame significant methodological limitations of previous studies using other *B. infantis* strains, such as lack of a control group, short intervention periods, and low sample sizes. In addition, we used a validated instrument (IGSQ) for assessing infant gastro-intestinal symptoms and included measures of selected fecal barrier and immune markers (i.e., cytokines). To the best of our knowledge, our study is the first probiotic intervention where infant gut microbiota is analyzed at strain level using shotgun metagenomics. Some study limitations to acknowledge include a relatively short follow-up period missing potential long-term immune and gut health benefits, the lack of coverage of geographies beyond Philippines, and lack of blood samples to allow understanding the impact on the host systemic immune status. In addition, the low proportion of C-section delivered infants (15%) did not allow an analysis stratified by delivery mode.

In conclusion, *B. infantis* LMG11588 supplementation, at low or high dose, was associated with normal infant growth, and was demonstrated to be safe and well-tolerated. It also promoted an infant-type *Bifidobacterium*-rich microbiota, driven by *B. infantis* LMG11588 colonization, without interfering with the ecology of the autochthonous *B. infantis* strains. Finally, in exclusively breastfed infants, *B. infantis* supplementation encouraged microbial metabolic activity and beneficially modulated enteric inflammation.

## Data availability statement

The original contributions presented in the study are included in the article/Supplementary material, further inquiries can be directed to the corresponding author.

## Ethics statement

The studies involving humans were approved by Institutional Review Board at Asian Hospital and Medical Centre Research Ethics Committee. The studies were conducted in accordance with the local legislation and institutional requirements. Written informed consent for participation in this study was provided by the participants’ legal guardians/next of kin.

## Author contributions

MC: Funding acquisition, Investigation, Project administration, Resources, Supervision, Writing – review & editing. LP: Investigation, Resources, Supervision, Writing – review & editing. CM: Data curation, Formal analysis, Visualization, Writing – original draft, Writing – review & editing. MN: Formal analysis, Investigation, Methodology, Resources, Supervision, Writing – original draft, Writing – review & editing. KV: Formal analysis, Investigation, Methodology, Supervision, Writing – original draft, Writing – review & editing. GLC: Formal analysis, Methodology. AF: Data curation, Formal analysis, Writing – review & editing. JM: Data curation, Formal analysis, Investigation, Software, Writing – original draft, Writing – review & editing. JV: Investigation, Software, Supervision, Writing – review & editing. PM: Methodology, Software, Writing – review & editing. BN: Conceptualization, Methodology, Resources, Software, Supervision, Writing – review & editing. CB: Writing – review & editing. TS: Project administration, Resources, Writing – review & editing. BB: Formal analysis, Investigation, Supervision, Writing – original draft, Writing – review & editing. CC: Conceptualization, Formal analysis, Investigation, Project administration, Resources, Supervision, Writing – original draft, Writing – review & editing.

## References

[1] Favier CF Vaughan EE De Vos WM Akkermans AD. Molecular monitoring of succession of bacterial communities in human neonates. Appl Environ Microbiol. (2002) 68:219–26. doi: 10.1128/AEM.68.1.219-226.2002, PMID: 11772630 PMC126580

[2] Jia Q Yu X Chang Y You Y Chen Z Wang Y. Dynamic changes of the gut microbiota in preterm infants with different gestational age. Front Microbiol. (2022) 13:923273. doi: 10.3389/fmicb.2022.923273, PMID: 35847070 PMC9279133

[3] Dominguez-Bello MG Costello EK Contreras M Magris M Hidalgo G Fierer N. Delivery mode shapes the acquisition and structure of the initial microbiota across multiple body habitats in newborns. Proc Natl Acad Sci U S A. (2010) 107:11971–5. doi: 10.1073/pnas.1002601107, PMID: 20566857 PMC2900693

[4] Guaraldi F Salvatori G. Effect of breast and formula feeding on gut microbiota shaping in newborns. Front Cell Infect Microbiol. (2012) 2:94. doi: 10.3389/fcimb.2012.00094 23087909 PMC3472256

[5] Ficara M Pietrella E Spada C Della Casa Muttini E Lucaccioni L Iughetti L. Changes of intestinal microbiota in early life. J Matern Fetal Neonatal Med. (2020) 33:1036–43. doi: 10.1080/14767058.2018.1506760 30058404

[6] Saturio S Nogacka AM Alvarado-Jasso GM Salazar N de los Reyes-Gavilán CG Gueimonde M. Role of Bifidobacteria on infant health. Microorganisms. (2021) 9:122415. doi: 10.3390/microorganisms9122415, PMID: 34946017 PMC8708449

[7] Laursen MF Sakanaka M von Burg N Mörbe U Andersen D Moll JM. *Bifidobacterium* species associated with breastfeeding produce aromatic lactic acids in the infant gut. Nat Microbiol. (2021) 6:1367–82. doi: 10.1038/s41564-021-00970-4, PMID: 34675385 PMC8556157

[8] Braegger C Chmielewska A Decsi T Kolacek S Mihatsch W Moreno L. Supplementation of infant formula with probiotics and/or prebiotics: a systematic review and comment by the ESPGHAN committee on nutrition. J Pediatr Gastroenterol Nutr. (2011) 52:238–50. doi: 10.1097/MPG.0b013e3181fb9e80 21150647

[9] Chichlowski M Shah N Wampler JL Wu SS Vanderhoof JA. *Bifidobacterium longum* subspecies *infantis* (*B. infantis*) in Pediatric nutrition: current state of knowledge. Nutrients. (2020) 12:1581. doi: 10.3390/nu12061581, PMID: 32481558 PMC7352178

[10] Stilling RM van de Wouw M Clarke G Stanton C Dinan TG Cryan JF. The neuropharmacology of butyrate: the bread and butter of the microbiota-gut-brain axis? Neurochem Int. (2016) 99:110–32. doi: 10.1016/j.neuint.2016.06.011, PMID: 27346602

[11] Parada Venegas D de la Fuente MK Landskron G González MJ Quera R Dijkstra G. Short chain fatty acids (SCFAs)-mediated gut epithelial and immune regulation and its relevance for inflammatory bowel diseases. Front Immunol. (2019) 10:277. doi: 10.3389/fimmu.2019.00277, PMID: 30915065 PMC6421268

[12] Henrick BM Chew S Casaburi G Brown HK Frese SA Zhou Y. Colonization by *B. infantis* EVC001 modulates enteric inflammation in exclusively breastfed infants. Pediatr Res. (2019) 86:749–57. doi: 10.1038/s41390-019-0533-2, PMID: 31443102 PMC6887859

[13] Frese SA Hutton AA Contreras LN Shaw CA Palumbo MC Casaburi G. Persistence of supplemented *Bifidobacterium longum* subsp. *infantis* EVC001 in breastfed infants. mSphere. (2017) 2:17. doi: 10.1128/mSphere.00501-17, PMID: 29242832 PMC5717325

[14] Taft DH Lewis ZT Nguyen N Ho S Masarweh C Dunne-Castagna V. *Bifidobacterium* species colonization in infancy: a global cross-sectional comparison by population history of breastfeeding. Nutrients. (2022) 14:1423. doi: 10.3390/nu14071423, PMID: 35406036 PMC9003546

[15] Ellis CL Bokulich NA Kalanetra KM Mirmiran M Elumalai J Haapanen L. Probiotic administration in congenital heart disease: a pilot study. J Perinatol. (2013) 33:691–7. doi: 10.1038/jp.2013.41, PMID: 23599119 PMC3758394

[16] Powell WT Borghese RA Kalanetra KM Mirmiran M Mills DA Underwood MA. Probiotic Administration in Infants with Gastroschisis: a pilot randomized placebo-controlled trial. J Pediatr Gastroenterol Nutr. (2016) 62:852–7. doi: 10.1097/MPG.0000000000001031, PMID: 26545203 PMC4854817

[17] Underwood MA Kalanetra KM Bokulich NA Lewis ZT Mirmiran M Tancredi DJ. A comparison of two probiotic strains of bifidobacteria in premature infants. J Pediatr. (2013) 163:1585–91. doi: 10.1016/j.jpeds.2013.07.017, PMID: 23993139 PMC3842430

[18] De Andres J Manzano S Garcia C Rodriguez JM Espinosa-Martos I Jimenez E. Modulatory effect of three probiotic strains on infants' gut microbial composition and immunological parameters on a placebo-controlled, double-blind, randomised study. Benef Microbes. (2018) 9:573–84. doi: 10.3920/BM2017.0132 29726280

[19] Manzano S De Andrés J Castro I Rodríguez JM Jiménez E Espinosa-Martos I. Safety and tolerance of three probiotic strains in healthy infants: a multi-Centre randomized, double-blind, placebo-controlled trial. Benef Microbes. (2017) 8:569–78. doi: 10.3920/BM2017.0009, PMID: 28555502

[20] Smilowitz JT Moya J Breck MA Cook C Fineberg A Angkustsiri K. Safety and tolerability of *Bifidobacterium longum* subspecies *infantis* EVC001 supplementation in healthy term breastfed infants: a phase I clinical trial. BMC Pediatr. (2017) 17:133. doi: 10.1186/s12887-017-0886-9, PMID: 28558732 PMC5450358

[21] Duboux S Ngom-Bru C De Bruyn F Phylogenetic BB. Functional and safety features of 1950s *B. infantis* strains. Microorganisms. (2022) 10:20203. doi: 10.3390/microorganisms10020203, PMID: 35208658 PMC8879182

[22] Riley AW Trabulsi J Yao M Bevans KB DeRusso PA. Validation of a parent report questionnaire: the infant gastrointestinal symptom questionnaire. Clin Pediatr. (2015) 54:1167–74. doi: 10.1177/0009922815574075, PMID: 25758425 PMC4564761

[23] Pouteau E Vahedi K Messing B Flourié B Nguyen P Darmaun D. Production rate of acetate during colonic fermentation of lactulose: a stable-isotope study in humans. Am J Clin Nutr. (1998) 68:1276–83. doi: 10.1093/ajcn/68.6.1276, PMID: 9846859

[24] Riva A Gray EH Azarian S Zamalloa A McPhail MJW Vincent RP. Faecal cytokine profiling as a marker of intestinal inflammation in acutely decompensated cirrhosis. JHEP Rep. (2020) 2:100151. doi: 10.1016/j.jhepr.2020.100151, PMID: 32838247 PMC7391986

[25] Bosheva M Tokodi I Krasnow A Pedersen HK Lukjancenko O Eklund AC. Infant formula with a specific blend of five human Milk oligosaccharides drives the gut microbiota development and improves gut maturation markers: a randomized controlled trial. Front Nutr. (2022) 9:920362. doi: 10.3389/fnut.2022.920362, PMID: 35873420 PMC9298649

[26] Holmes I Harris K Quince C. Dirichlet multinomial mixtures: generative models for microbial metagenomics. PLoS One. (2012) 7:e30126. doi: 10.1371/journal.pone.0030126, PMID: 22319561 PMC3272020

[27] O'Leary NA Wright MW Brister JR Ciufo S Haddad D McVeigh R. Reference sequence (ref Seq) database at NCBI: current status, taxonomic expansion, and functional annotation. Nucleic Acids Res. (2016) 44:D733–45. doi: 10.1093/nar/gkv1189, PMID: 26553804 PMC4702849

[28] Li H. Improving SNP discovery by base alignment quality. Bioinformatics. (2011) 27:1157–8. doi: 10.1093/bioinformatics/btr076, PMID: 21320865 PMC3072548

[29] Li H. A statistical framework for SNP calling, mutation discovery, association mapping and population genetical parameter estimation from sequencing data. Bioinformatics. (2011) 27:2987–93. doi: 10.1093/bioinformatics/btr509, PMID: 21903627 PMC3198575

[30] Nguyen LT Schmidt HA von Haeseler A Minh BQ. IQ-TREE: a fast and effective stochastic algorithm for estimating maximum-likelihood phylogenies. Mol Biol Evol. (2015) 32:268–74. doi: 10.1093/molbev/msu300, PMID: 25371430 PMC4271533

[31] Chernomor O von Haeseler A Minh BQ. Terrace aware data structure for phylogenomic inference from supermatrices. Syst Biol. (2016) 65:997–1008. doi: 10.1093/sysbio/syw037, PMID: 27121966 PMC5066062

[32] Kalyaanamoorthy S Minh BQ Wong TKF von Haeseler A Jermiin LS. Model finder: fast model selection for accurate phylogenetic estimates. Nat Methods. (2017) 14:587–9. doi: 10.1038/nmeth.4285, PMID: 28481363 PMC5453245

[33] Jones P Binns D Chang HY Fraser M Li W McAnulla C. Inter pro scan 5: genome-scale protein function classification. Bioinformatics. (2014) 30:1236–40. doi: 10.1093/bioinformatics/btu031, PMID: 24451626 PMC3998142

[34] Katoh K Standley DM. MAFFT multiple sequence alignment software version 7: improvements in performance and usability. Mol Biol Evol. (2013) 30:772–80. doi: 10.1093/molbev/mst010, PMID: 23329690 PMC3603318

[35] Katoh K Misawa K Kuma K Miyata T. MAFFT: a novel method for rapid multiple sequence alignment based on fast Fourier transform. Nucleic Acids Res. (2002) 30:3059–66. doi: 10.1093/nar/gkf436, PMID: 12136088 PMC135756

[36] Joensen KG Engsbro A Lukjancenko O Kaas RS Lund O Westh H. Evaluating next-generation sequencing for direct clinical diagnostics in Diarrhoeal disease. Eur J Clin Microbiol Infect Dis. (2017) 36:1325–38. doi: 10.1007/s10096-017-2947-2, PMID: 28285331 PMC5495851

[37] Barer Miwlsapn. Medical microbiology: A guide to microbial infections: Pathogenesis, immunity, laboratory investigation and control. (2019)

[38] Awad MM Johanesen PA Carter GP Rose E Lyras D. *Clostridium difficile* virulence factors: insights into an anaerobic spore-forming pathogen. Gut Microbes. (2014) 5:579–93. doi: 10.4161/19490976.2014.969632, PMID: 25483328 PMC4615314

[39] Freedman JC Shrestha A McClane BA. *Clostridium perfringens* enterotoxin: action, genetics, and translational applications. Toxins. (2016) 8:73. doi: 10.3390/toxins8030073, PMID: 26999202 PMC4810218

[40] Kenny B Warawa J. Enteropathogenic *Escherichia coli* (EPEC) Tir receptor molecule does not undergo full modification when introduced into host cells by EPEC-independent mechanisms. Infect Immun. (2001) 69:1444–53. doi: 10.1128/IAI.69.3.1444-1453.2001, PMID: 11179311 PMC98040

[41] Khaertynov KS Anokhin VA Rizvanov AA Davidyuk YN Semyenova DR Lubin SA. Virulence factors and antibiotic resistance of *Klebsiella pneumoniae* strains isolated from neonates with sepsis. Front Med. (2018) 5:225. doi: 10.3389/fmed.2018.00225, PMID: 30155466 PMC6102385

[42] Yeh KM Kurup A Siu LK Koh YL Fung CP Lin JC. Capsular serotype K1 or K2, rather than mag a and rmp a, is a major virulence determinant for *Klebsiella pneumoniae* liver abscess in Singapore and Taiwan. J Clin Microbiol. (2007) 45:466–71. doi: 10.1128/JCM.01150-06 17151209 PMC1829066

[43] Kaur J Jain SK. Role of antigens and virulence factors of *Salmonella enterica* serovar Typhi in its pathogenesis. Microbiol Res. (2012) 167:199–210. doi: 10.1016/j.micres.2011.08.001, PMID: 21945101

[44] Clausen P Aarestrup FM Lund O. Rapid and precise alignment of raw reads against redundant databases with KMA. BMC Bioinf. (2018) 19:307. doi: 10.1186/s12859-018-2336-6, PMID: 30157759 PMC6116485

[45] Estorninos E Lawenko RB Palestroque E Lebumfacil J Marko M Cercamondi CI. Infant formula containing bovine milk-derived oligosaccharides supports age-appropriate growth and improves stooling pattern. Pediatr Res. (2022) 91:1485–92. doi: 10.1038/s41390-021-01541-3, PMID: 33958719 PMC9197766

[46] Pediatrics AAo, Clinical testing of infant formulas with respect to nutritional suitability for term infants. Report to the FDA. (1988)

[47] R: A language and environment for statistical computing [computer program]. Vienna, Austria: Foundation for Statistical Computing (2022).

[48] Vatanen T Ang QY Siegwald L Sarker SA le Roy CI Duboux S. A distinct clade of *Bifidobacterium longum* in the gut of Bangladeshi children thrives during weaning. Cells. (2022) 185:4280. doi: 10.1016/j.cell.2022.10.011, PMID: 36323316

[49] Dupont C Rivero M Grillon C Belaroussi N Kalindjian A Marin V. α-Lactalbumin-enriched and probiotic-supplemented infant formula in infants with colic: growth and gastrointestinal tolerance. Eur J Clin Nutr. (2010) 64:765–7. doi: 10.1038/ejcn.2010.81, PMID: 20517331

[50] Zhang S Li T Xie J Zhang D Pi C Zhou L. Gold standard for nutrition: a review of human milk oligosaccharide and its effects on infant gut microbiota. Microb Cell Factories. (2021) 20:108. doi: 10.1186/s12934-021-01599-y, PMID: 34049536 PMC8162007

[51] Sánchez C Fente C Regal P Lamas A Lorenzo MP. Human Milk oligosaccharides (HMOs) and infant microbiota: a scoping review. Foods. (2021) 10. doi: 10.3390/foods10061429, PMID: 34203072 PMC8234547

[52] Walsh C Lane JA van Sinderen D Hickey RM. Human milk oligosaccharides: shaping the infant gut microbiota and supporting health. J Funct Foods. (2020) 72:104074. doi: 10.1016/j.jff.2020.104074, PMID: 32834834 PMC7332462

[53] Bode L. The functional biology of human milk oligosaccharides. Early Hum Dev. (2015) 91:619–22. doi: 10.1016/j.earlhumdev.2015.09.001 26375354

[54] Walter J Ley R. The human gut microbiome: ecology and recent evolutionary changes. (2011), 65, 411–429, doi: 10.1146/annurev-micro-090110-102830 21682646

[55] Norris RF Flanders T Tomarelli R György P. The isolation and cultivation of lactobacillus bifidus: a comparison of branched and unbranched strains. J Bacteriol. (1950) 60:681–96. doi: 10.1128/jb.60.6.681-696.1950, PMID: 14824062 PMC385940

[56] Barratt MJ Nuzhat S Ahsan K Frese SA Arzamasov AA Sarker SA. *Bifidobacterium infantis* treatment promotes weight gain in Bangladeshi infants with severe acute malnutrition. Sci Transl Med. (2022) 14:eabk1107. doi: 10.1126/scitranslmed.abk1107, PMID: 35417188 PMC9516695

[57] Arrieta M-C Arévalo A Stiemsma L. Associations between infant fungal and bacterial dysbiosis and childhood atopic wheeze in a nonindustrialized setting. J Allergy Clin Immunol. (2018) 142:424–434.e10. doi: 10.1016/j.jaci.2017.08.041, PMID: 29241587 PMC6075469

[58] Arrieta M Stiemsma L Dimitriu P. Early infancy microbial and metabolic alterations affect risk of childhood asthma. Sci Transl Med. (2015) 7:307ra152. doi: 10.1126/scitranslmed.aab2271 26424567

[59] Vatanen T Kostic AD d’Hennezel E Siljander H Franzosa EA Yassour M. Variation in microbiome LPS immunogenicity contributes to autoimmunity in humans. Cells. (2016) 165:842–53. doi: 10.1016/j.cell.2016.04.007, PMID: 27133167 PMC4950857

[60] Sonnenburg ED Sonnenburg JL. The ancestral and industrialized gut microbiota and implications for human health. Nat Rev Microbiol. (2019) 17:383–90. doi: 10.1038/s41579-019-0191-8, PMID: 31089293

[61] Henrick BM Rodriguez L Lakshmikanth T Pou C Henckel E Arzoomand A. Bifidobacteria-mediated immune system imprinting early in life. Cells. (2021) 184:3884–98. doi: 10.1016/j.cell.2021.05.030, PMID: 34143954

[62] Underwood MA German JB Lebrilla CB Mills DA. *Bifidobacterium longum* subspecies *infantis*: champion colonizer of the infant gut. Pediatr Res. (2015) 77:229–35. doi: 10.1038/pr.2014.156, PMID: 25303277 PMC4350908

