# Supplementary material for: Safety, efficacy, and impact on gut microbial ecology of a Bifidobacterium longum subspecies infantis LMG11588 supplementation in healthy term infants: a randomized, double-blind, controlled trial in the Philippines
Source: Front Nutr. 2023 Dec 14;10:1319873. doi: 10.3389/fnut.2023.1319873 (PMC10755859; doi:10.3389/fnut.2023.1319873)
Supplement: Supplementary file 1 [file Data_Sheet_1.PDF]

Supplementary methods

Participants

Exclusion criteria were as follows: infants with conditions requiring infant feedings other than those specified in the protocol; infants receiving complementary foods or liquids defined as 4 or more teaspoons per day or approximately 20 grams per day; infants with a medical condition or history that could increase the risk associated with study participation or interfere with the interpretation of study results (including evidence of major congenital malformations; suspected or documented systemic or congenital infections; history of admission to the Neonatal Intensive Care Unit, except for admission for jaundice phototherapy; other severe medical or laboratory abnormality); infants receiving or who previously received probiotic supplements or any medication(s) or supplement(s) which are known or suspected to affect the following: fat digestion, absorption, and/or metabolism; stool characteristics; growth; gastric acid secretion.

Safety and tolerance outcomes

Infant weight was assessed on a calibrated scale without clothing or diaper, and weight was recorded to the nearest 1 g. Recumbent length was measured on a standardized length board and recorded to the nearest 0.1 cm. Head circumference was measured at the maximum circumference of the head using a standard non-elastic plastic-coated measuring tape and recorded to the nearest 0.1 cm. Corresponding z-scores for weight, length, and head circumference were determined (weight-for-age, length-for-age, weight-for-length, and head circumference-for-age) using the WHO Child Growth Standards as the reference population[1]. Categorical measures were used for frequency of regurgitation/vomiting (0 times, 1 time, 2–3 times, or 4–6 times per day), and frequency of crying and flatulence episodes (0 times, 1 time, 2-3 times per day). Stool frequency was calculated as the mean number of stools reported retrospectively over a 1-day period at baseline or prospectively over the 3-day periods just prior to V2 and V3. Difficulty in passing stool was counted as the number of stools difficult to pass per day from each set of diaries. Stool consistency was reported using a validated 5-point stool scale developed for infants ≤ one year of age (1=watery, 2=runny, 3=mushy/soft, 4=firm,

5=hard)[2] and mean stool consistency was calculated for stools reported at baseline or just prior to V2 and V3.

We used the validated Infant Gastrointestinal Symptom Questionnaire-13 (IGSQ) which includes 13 questions covering 5 domains (stooling, vomiting/spit-up, crying, fussiness, and flatulence)[3]. Scores from each question are summed to calculate an overall index score which can range from 13 (low GI burden) to 65 (high GI burden).

Microbiome analysis and ecological measures

Briefly, microbial DNA was extracted from frozen feces, purified, sheared unto 350 bp fragments, and shotgun sequenced with 2 × 150 bp paired-end sequencing on an Illumina NovaSeq 6000 platform. After removal of low-quality reads and reads aligning to the human genome (GRCh38), reads were aligned to the Clinical Microbiomics in-house extended infant fecal microbiome gene catalog (containing 20,992,486 microbial genes) allowing for taxonomical profiling of corresponding set of 1472 MGS[4]. Alpha and beta diversity estimates were calculated from rarefied abundance matrices created by random sampling of reads without replacement. A phylogenetic tree connecting the MGSs was generated using previously identified single-copy marker genes and used for calculation of phylogenetic diversity measures. Alpha diversity was calculated on gene, MGS, and genus level and as MGS-level Faith's phylogenetic diversity (PD)[5] using the PhyloMeasures R package. Beta diversity was calculated as Bray-Curtis dissimilarity and weighted UniFrac distance[6] (using the phyloseq R package). Detection of species with pathogenic potential was done by mapping species-specific virulence factors present in samples to MGSs.[7-10]

References

1. World Health Organization. *WHO child growth standards: length/height-for-age, weight-for-age, weight-for-length, weight-for-height and body mass index-for-age: methods and development.* 2006.
2. Weaver LT, Ewing G, Taylor LC. The bowel habit of milk-fed infants. *J Pediatr Gastroenterol Nutr.* 1988;7(4):568-571.
3. Riley AW, Trabulsi J, Yao M, Bevans KB, DeRusso PA. Validation of a Parent Report Questionnaire: The Infant Gastrointestinal Symptom Questionnaire. *Clin Pediatr (Phila).* 2015;54(12):1167-1174.
4. Nielsen HB, Almeida M, Juncker AS, et al. Identification and assembly of genomes and genetic elements in complex metagenomic samples without using reference genomes. *Nat Biotechnol.* 2014;32(8):822-828.

5. Faith DP, Baker AM. Phylogenetic diversity (PD) and biodiversity conservation: some bioinformatics challenges. *Evol Bioinform Online.* 2007;2:121-128.
6. Hamady M, Lozupone C, Knight R. Fast UniFrac: facilitating high-throughput phylogenetic analyses of microbial communities including analysis of pyrosequencing and PhyloChip data. *Isme j.* 2010;4(1):17-27.
7. Parks DH, Imelfort M, Skennerton CT, Hugenholtz P, Tyson GW. CheckM: assessing the quality of microbial genomes recovered from isolates, single cells, and metagenomes. *Genome Res.* 2015;25(7):1043-1055.
8. Langmead B, Salzberg SL. Fast gapped-read alignment with Bowtie 2. *Nat Methods.* 2012;9(4):357-359.
9. Schubert M, Lindgreen S, Orlando L. AdapterRemoval v2: rapid adapter trimming, identification, and read merging. *BMC Res Notes.* 2016;9:88.
10. Li H, Durbin R. Fast and accurate short read alignment with Burrows-Wheeler transform. *Bioinformatics.* 2009;25(14):1754-1760.
