# Supplementary figures and images for: Safety, efficacy, and impact on gut microbial ecology of a Bifidobacterium longum subspecies infantis LMG11588 supplementation in healthy term infants: a randomized, double-blind, controlled trial in the Philippines
Source: Front Nutr. 2023 Dec 14;10:1319873. doi: 10.3389/fnut.2023.1319873 (PMC10755859; doi:10.3389/fnut.2023.1319873)

Supplementary Figure 1

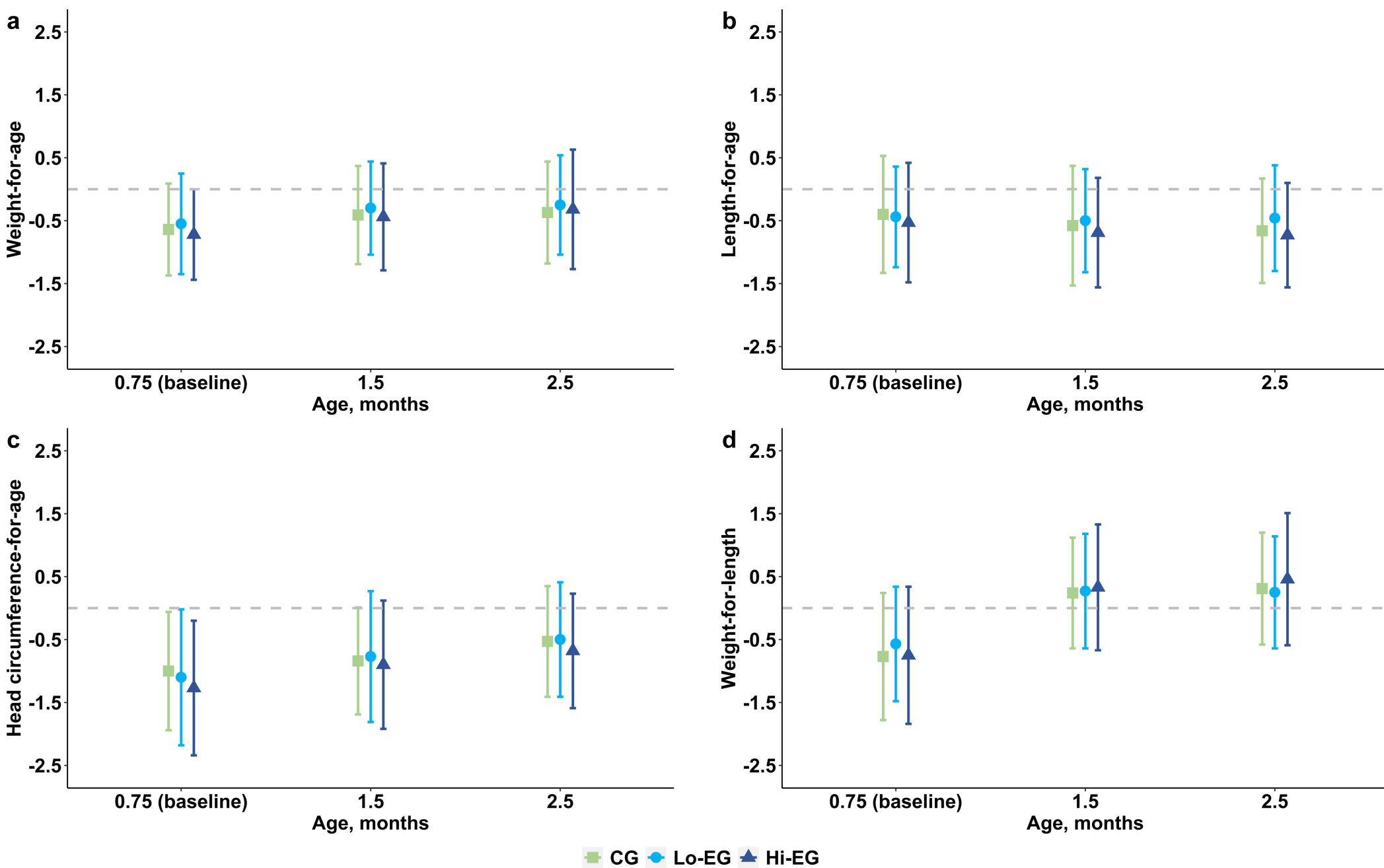

Supplementary Figure 2

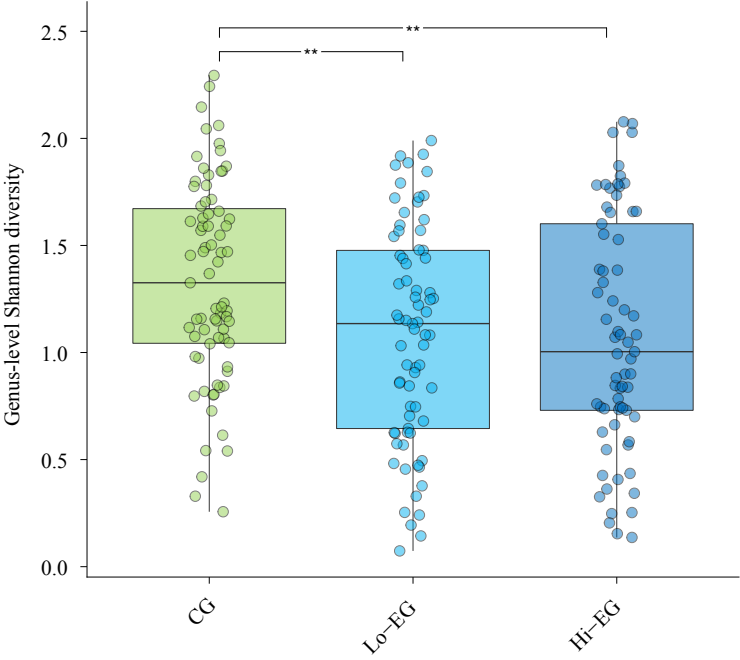

Supplementary Figure 3

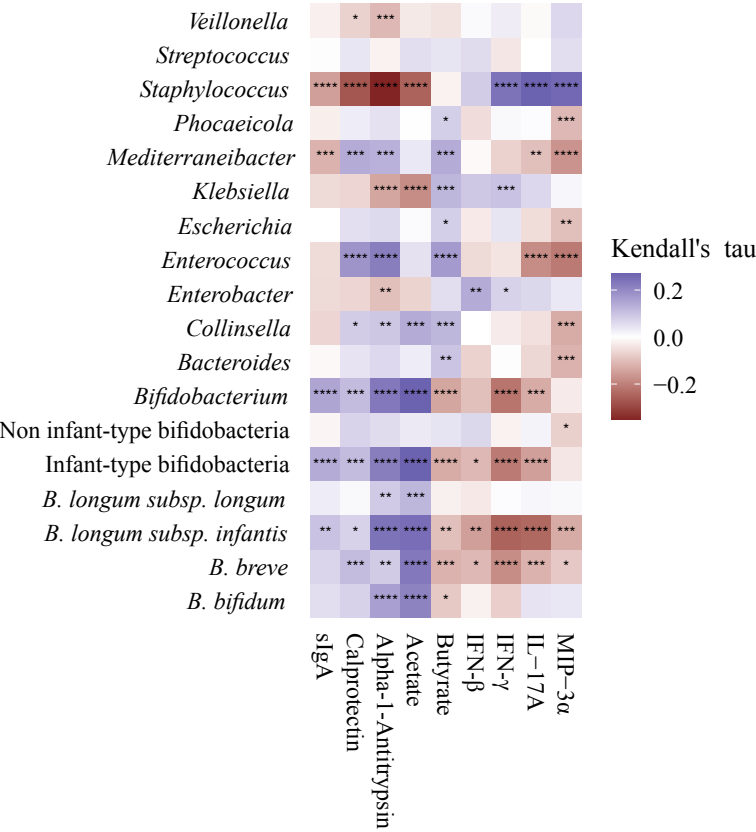

Supplementary Figure 4

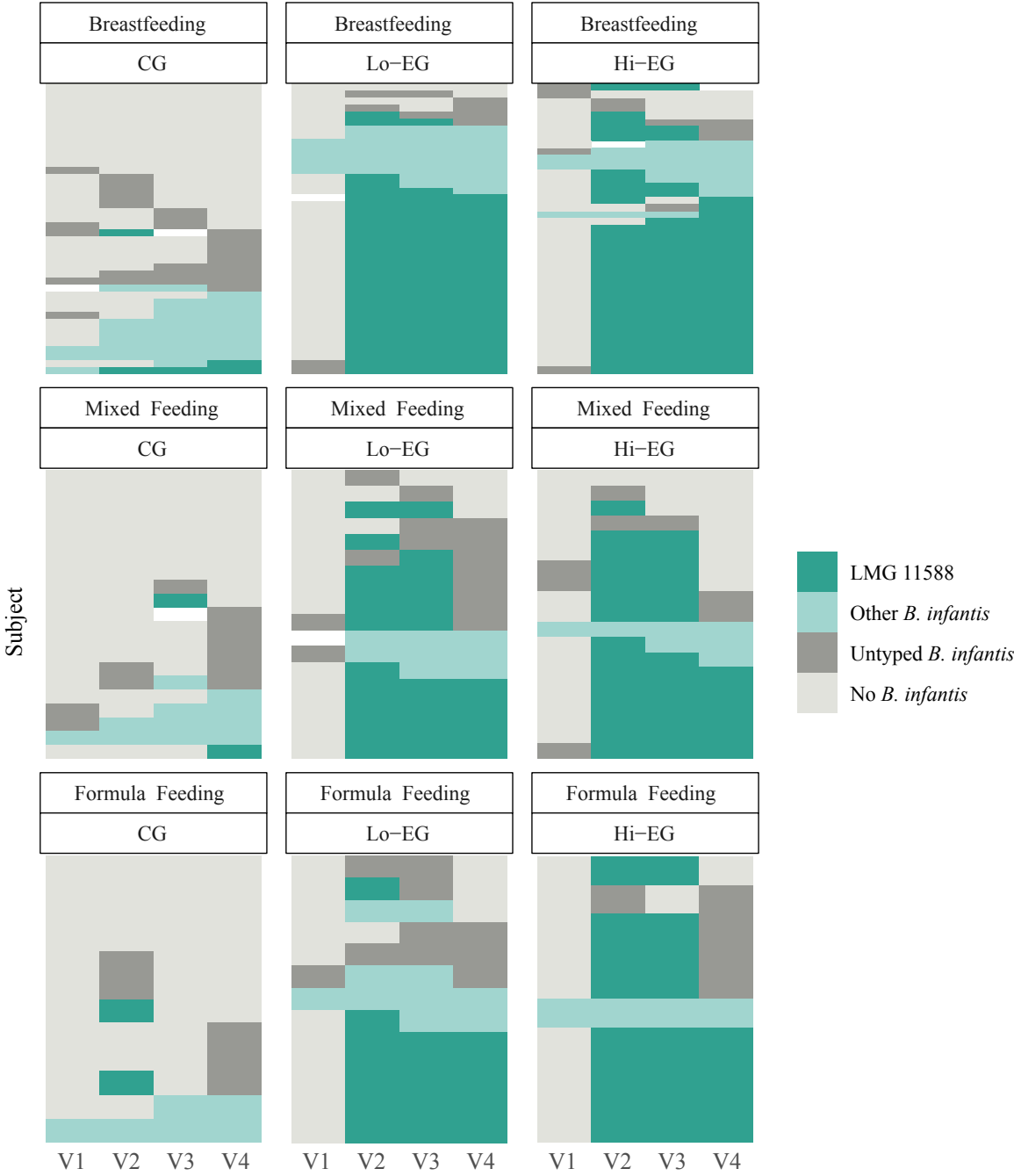

Supplement: Supplementary file 2 [file Data_Sheet_2.PDF]
