# Supplementary material for: Safety, efficacy, and impact on gut microbial ecology of a Bifidobacterium longum subspecies infantis LMG11588 supplementation in healthy term infants: a randomized, double-blind, controlled trial in the Philippines
Source: Front Nutr. 2023 Dec 14;10:1319873. doi: 10.3389/fnut.2023.1319873 (PMC10755859; doi:10.3389/fnut.2023.1319873)
Supplement: Supplementary file 3 [file Data_Sheet_3.pdf]

Supplementary Table 1. Descriptive statistics and adjusted comparisons of IGSQ-13 score among groups and by visit, FAS

Visit	Group	Geometric means (SD)	Estimate[a] (95% CI)	Group comparisons: Comparison	Group comparisons: Ratio of estimate (95% CI)	Group comparisons: p-value[b]
Baseline	CG	14·7 (1·1)	n/a	n/a	n/a	n/a
	Lo-EG	14·5 (1·1)	n/a	n/a	n/a	n/a
	Hi-EG	14·5 (1·1)	n/a	n/a	n/a	n/a
Visit 2	CG	14·7 (1·1)	14·6 (14·2, 15·1)	--	--	--
	Lo-EG	14·7 (1·1)	14·7 (14·3, 15·1)	Lo-EG vs CG	1·00 (0·96,1·04)	0·944
	Hi-EG	14·8 (1·2)	14·9 (14·4, 15·3)	Hi-EG vs CG	1·01 (0·97, 1·06))	0·806
Visit 3	CG	14·0 (1·1)	13·9 (13·7, 14·2)	--	--	--
	Lo-EG	13·9 (1·1)	13·9 (13·7, 14·2)	Lo-EG vs CG	1·00 (0·97, 1·03)	0·991
	Hi-EG	13·9 (1·1)	13·9 (13·6, 14·2)	Hi-EG vs CG	1·00 (0·97, 1·03)	0·991

ANCOVA, analysis of covariance; CG, control group (placebo supplement); FAS, full analysis set; IGSQ, Infant Gastrointestinal Symptom Questionnaire (IGSQ); Lo-EG, low-dose *B. infantis*,1·0 x 10^8 CFU; Hi-EG, high-dose *B. infantis*, 1·8 x 10^{10} CFU; SD, standard deviation; CI, confidence interval

[a] From an ANCOVA model correcting for baseline weight and sex.

[b] Adjusted p-values were calculated using Benjamini-Hochberg correction.

Supplementary Table 2. GI tolerance outcomes by visit, FAS

Visit	Category	CG (n=75)	Lo-EG (n=73)	Hi-EG ((n=70)	Lo-EG v CG p-value[d]	Hi-EG v CG p-value[d]	Hi-EG v Lo-EG p-value[d]
**Stool frequency[a,b], LS mean (95% CI)**							
Visit 2		1·9 (1·6, 2·2)	1·9 (1·6, 2·2)	1·9 (1·6, 2·2)	0·943	0·943	0·943
Visit 3		1·7 (1·5, 2·0)	1·6 (1·3, 1·9)	1·5 (1·2, 1·8)	0·576	0·576	0·576
**Stool consistency score[a,b], LS mean (95% CI)**							
Visit 2		2·9 (2·8, 3·0)	2·9 (2·8, 3·0)	2·8 (2·7, 2·9)	0·742	0·155	0·161
Visit 3		2·8 (2·7, 3·0)	2·8 (2·7, 2·9)	2·8 (2·6, 2·9)	0754	0·754	0·754
**Stool consistency[a], N stools (%)**							
Visit 2	Watery	3 (0·7%)	4 (1·0%)	19 (4·9%)	**0·021**	**<0·0001**	**<0·0001**
	Runny	82 (18·9%)	76 (18·4%)	103 (26·4%)			
	Soft	299 (68·7%)	309 (75·0%)	241 (61·8%)			
	Formed	48 (11·0%)	21 (5·1%)	27 (6·9%)			
	Hard	3 (0·7%)	2 (0·5%)	0 (0%)			
Visit 3	Watery	12 (3·1%)	8 (2·3%)	14 (4·5%)	0.490	0 ·308	0·490
	Runny	73 (18·7%)	79 (22·6%)	74 (23·8%)			
	Soft	268 (68·7%)	235 (67·3%)	204 (65·6%)			
	Formed	33 (8·5%)	26 (7·4%)	19 (6·1%)			
	Hard	4 (1·0%)	1 (0·3%)	0 (0%)			
**Difficulty passing stool[a,c], N stools (%)**							
Visit 2		6 (2·7%)	4 (1·8%)	2 (1·0%)	0 ·513	0·495	0·513
Visit 3		2 (0·9%)	1 (0·5%)	1 (0·5%)	0·915	0·915	0·956
**Regurgitation/Vomiting, N reports (%)**							
Visit 2	0 times	97 (43·1%)	99 (45·2%)	117 (55·7%)	0·125	**0·031**	0·052
	1 time	31 (13·8%)	42 (19·2%)	32 (15·2%)			
	2-3 times	72 (32·0%)	67 (30·6%)	46 (21·9%)			
	4-6 times	20 (8·9%)	9 (4·1%)	15 (7·1%)			
	7 or more	5 (2·2%)	2 (0·9%)	0 (0%)			

Visit	Category	CG (n=75)	Lo-EG (n=73)	Hi-EG ((n=70)	Lo-EG v CG p-value[d]	Hi-EG v CG p-value[d]	Hi-EG v Lo-EG p-value[d]
**Regurgitation/Vomiting con't, N reports (%)**							
Visit 3	0 times	142 (63·1%)	146 (66·7%)	136 (64·8%)	**0·006**	**0·044**	**0·006**
	1 time	26 (11·6%)	17 (7·8%)	36 (17·1%)			
	2-3 times	43 (19·1%)	55 (25·1%)	35 (16·7%)			
	4-6 times	12 (5·3%)	1 (0·5%)	3 (1·4%)			
	7 or more	2 (0·9%)	0 (0%)	0 (0%)			
**Flatulence, N reports (%)**							
Visit 2	0 times	171 (76·0%)	182 (83·1%)	156 (74·3%)	0·087	0·928	0·087
	1 time	15 (6·7%)	15 (6·8%)	18 (8·6%)			
	2-3 times	32 (14·2%)	15 (6·8%)	31 (14·8%)			
	4-6 times	5 (2·2%)	7 (3·2%)	4 (1·9%)			
	7 or more	2 (0·9%)	0 (0%)	1 (0·5%)			
Visit 3	0 times	200 (88·9%)	200 (91·3%)	180 (85·7%)	0·141	0.141	0·243
	1 time	7 (3·1%)	11 (5·0%)	17 (8·1%)			
	2-3 times	18 (8·0%)	8 (3·7%)	12 (5·7%)			
	4-6 times	0 (0%)	0 (0%)	1 (0·5%)			
	7 or more	0 (0%)	0 (0%)	0 (0%)			
**Crying/fussiness, N reports (%)**							
Visit 2	0 times	224 (99·6%)	212 (96·8%)	205 (97·6%)	0·066	0·133	0·876
	1 time	1 (0·4%)	1 (0·5%)	1 (0·5%)			
	2-3 times	0 (0%)	6 (2·7%)	4 (1·9%)			
Visit 3	0 times	225 (100·0%)	217 (99·1%)	210 (100·0%)	0·485	n/a	0·499
	1 time	0 (0%)	0 (0%)	0 (0%)			
	2-3 times	0 (0%)	2 (0·9%)	0 (0%)			

CG, control group (placebo supplement); Lo-EG, low-dose *B. infantis*,1.0 x 10^8 CFU; Hi-EG, high-dose *B. infantis*, 1.8 x 10^{10} CFU; LS mean, least squares mean; CI, confidence interval. Includes all measurements in the 1 day prior to Visit 1 and in the 3-day period prior to Visits 2 and 3.

[a] Data were analyzed by ANCOVA and adjusted for baseline values.

[b] Average scores over the 1 day prior to baseline, or the three days prior to V2 and V3 were analyzed.

[c] Calculated as the percentage of days with difficulties passing stool divided by total number of days on study.

[d]Adjusted p-values were calculated using Benjamini-Hochberg correction.

Supplementary Table 3. Physician-reported adverse events, FAS

	CG (n=75)		Lo-EG (n=73)		Hi-EG (n=70)	
	No. Events	N (%)	No. Events	N (%)	No. Events	N (%)
**All adverse events**	38	32 (42·7%)	33	27 (37·0%)	34	27 (38·6%)
**Serious adverse events**	1	1 (1.3%)	0	0 (0%)	0	0 (0%)
**Severity of Adverse Events**						
Mild	35	31 (41·3%)	32	27 (37·0%)	33	27 (38·6%)
Moderate	1	1 (1·3%)	1	1 (1·4%)	1	1 (1·4%)
Severe	2	1 (1·3%)	0	0 (0%)	0	0 (0%)
**Adverse events of interest, N (%)**						
Upper respiratory infection	9 (12·0%)		3 (4·1%)		6 (8·6%)	
Lower respiratory infection	6 (8·0%)		5 (6·8%)		7 (10·0%)	
Otitis/ear infection	0 (0%)		0 (0%)		0 (0%)	
Skin allergy	1 (1·3%)		0 (0%)		0 (0%)	
GI-related/diarrhea	0 (0%)		1 (1·4%)		0 (0%)	

CG, control group (placebo supplement); Lo-EG, low-dose *B. infantis*,1·0 x 10^8 CFU; Hi-EG, high-dose *B. infantis*, 1·8 x 10^{10} CFU

Supplementary Table 4. Parent reported symptoms, FAS

Symptom	CG (n=75) N (%)	Lo-EG (n=73) N (%)	Hi-EG1 (n=70) N (%)	Lo-EG vs CG p-value	Hi-EG vs CG p-value	Hi-EG vs Lo-EG p-value
**Fever**[a]	16 (21·3%)	23 (31·5%)	18 (25·7%)		0·369	
Duration[b]						
1 day	9 (12·0%)	13 (17·8%)	7 (10·0%)			
2 days	2 (2·7%)	6 (8·2%)	4 (5·7%)	0·746	0·746	>0·99
3 days	1 (1·3%)	0 (0·0%)	0 (0·0%)			
**Fever**[a]**, excluding vaccine-induced fever**[1]	5 (6·7%)	7 (9·6%)	8 (11·4%)		0·604	
Duration[b]						
1 day	1 (1·3%)	3 (4·1%)	1 (1·4%)	>0·99	>0·99	>0·99
2 days	0 (0%)	1 (1·4%)	2 (2·9%)			
**Respiratory tract symptoms**[a,c]	17 (22·7%)	7 (9·6%)	10 (14·3%)		0·085	
Duration[b]						
2 days	2 (2·7%)	1 (1·4%)	0 (0%)	>0·99	0·705	0·705
3 days	5 (6·7%)	3 (4·1%)	5 (7·1%)			
**Gastrointestinal symptoms**[a]	0 (0%)	1 (1·4%)	1 (1·4%)		0·976	
Duration[b]						
3 days	0 (0%)	1 (1·4%)	1 (1·4%)	n/a	n/a	n/a

CG, control group (placebo supplement); Lo-EG, low-dose *B. infantis*,1·0 x 10^8 CFU; Hi-EG, high-dose *B. infantis*, 1·8 x 10^{10} CFU.

[a] p-value from chi-square test comparing proportions between groups

[b] p-value from Fisher-Halton test comparing duration between pairwise groups. Adjusted using Benjamini-Hochberg correction. Symptom duration was available for the subset of symptoms confirmed by a PI.

[c] Predefined symptoms include cough, nasal congestion, sneezing, cackles/rales/wheezing, fever, difficulty breathing, hoarseness, rapid breathing, noisy breathing, and shallow breathing.

Supplementary Table 5a. Fecal pH (wet feces) and organic acids (dry feces, umol/g) at visits 2 and 3, FAS population (all subjects)

					Lo-EG v CG		Hi-EG v CG		Hi-EG vs Lo-EG	
**Marker**	**Visit**	**CG (n=75)**	**Lo-EG (n=73)**	**Hi-EG (n=70)**	**Unadj p-value**	**Adj p-value**	**Unadj p-value**	**Adj p-value**	**Unadj p-value**	**Adj p-value**
Fecal pH	Visit 2	5·38 (5·19, 5·57)	5·42 (5 ·22, 5 ·61)	5·10 (4·90, 5 ·30)	0·78	0·78	**0·051**	0·076	**0·027**	0·076
	Visit 3	5·.31 (5·06, 5·57)	5·36 (5·10, 5·61)	5·52 (5·25, 5·78)	0·82	0·82	0·28	0·59	0·39	0·59
Acetate	Visit 2	354·6 (313·6, 395·6)	406·3 (365·3, 447·3)	456·2 (414·3, 498·1)	0·080	0·095	**0·0008**	**0·0023**	0·095	0·095
	Visit 3	437·1 (388·8, 485·5)	481·5 (433·9, 529·1)	469·7 (420.3, 519.2)	0·20	0·53	0·35	0·53	0·74	0·74
Butyrate	Visit 2	26·3 (16·4, 36·3)	38·6 (28·7, 48·6)	31·5 (21·4, 41·7)	0·086	0·26	0·47	0·47	0·32	0·47
	Visit 3	33·1 (22·7, 43·4)	42·2 (32·0, 52·4)	42·8 (32·2, 53·4)	0·22	0·32	0·20	0·32	0·94	0·94
Isobutyrate	Visit 2	5·91 (3·97, 7·85)	5·89 (3·97, 7·81)	5·85 (3·71, 8·00)	0·99	0·99	0·97	0·99	0·98	0·99
	Visit 3	7·19 (4·87, 9·52)	5·68 (3·58, 7·78)	6·96 (4·78, 9·14)	0·34	0·61	0·89	0·89	0·41	0·61
Isovalerate	Visit 2	2·10 (1·52, 2·69)	2·52 (1·94, 3·11)	1·90 (1·31, 2·50)	0·32	0·47	0·64	0·64	0·14	0·43
	Visit 3	2·53 (1·70, 3·35)	3·28 (2·46, 4·09)	2·88 (2·03, 3·72)	0·21	0·56	0·56	0·56	0·50	0·56
Propionate	Visit 2	71·4 (57·1, 85·7)	68·5 (54·2, 82·8)	65·0 (50·3, 79·6)	0·78	0 ·78	0·53	0·78	0·73	0·78
	Visit 3	81·0 (63·0, 99·0)	80·8 (63·1, 98·6)	87·4 (69·0, 105·8)	0 ·99	0·99	0·63	0·94	0 ·62	0·94
Valerate	Visit 2	2·61 (1·37, 3·84)	4·37 (3·13, 5·60)	3·18 (1·91, 4·44)	**0·048**	0·14	0 ·52	0·52	0·19	0·28
	Visit 3	2·61 (1·37, 3·84)	4·37 (3·13, 5·60)	5·43 (3·71, 7·16)	0·42	0·53	0·16	0·47	0·53	0·53

CG, control group (placebo supplement); Lo-EG, low-dose *B. infantis*,1·0 x 10^8 CFU; Hi-EG, high-dose *B. infantis*, 1·8 x 10^{10} CFU. Data presented are LS mean (95% Confidence Interval) from ANCOVA model adjusted for baseline value. P-value from ANCOVA adjusted for baseline value. P-values are uncorrected.

Supplementary Table 5b. Fecal pH (wet feces) and organic acids (dry feces, umol/g) at visits 2 and 3, FAS population (breastfed only subjects)

					**Lo-EG v CG**		**Hi-EG v CG**		**Hi-EG vs Lo-EG**	
**Marker**	**Visit**	**CG (n=42)**	**Lo-EG (n=42)**	**Hi-EG (n=41)**	**Unadj p-value**	**Adj p-value**	**Unadj p-value**	**Adj p-value**	**Unadj p-value**	**Adj p-value**
Fecal pH	Visit 2	5·21 (4·99, 5·42)	5·03 (4·81, 5·24)	4·88 (4·67, 5·10)	0·24	0·36	**0·037**	0·12	0·36	0·36
	Visit 3	5·06 (4·75, 5·37)	4·96 (4·65, 5·27)	5·15 (4·84, 5·46)	0·64	0·69	0·69	0·69	0·39	0·69
Acetate	Visit 2	339·2 (283·0, 395·3)	414·1 (358·7, 469·6)	467·0 (410·1, 523·8)	0·063	0·094	**0·002**	**0·0059**	0·19	0·19
	Visit 3	394·6 (328·6, 460·5)	498·3 (434·5, 562·1)	490·8 (425·3, 556·4)	**0·028**	0·066	**0·044**	0·066	0·87	0·87
Butyrate	Visit 2	24·6 (13·6, 35·6)	23·8 (12·9, 34·7)	26·0 (14·9, 37·2)	0·92	0·92	0·85	0·92	0·78	0·92
	Visit 3	28·5 (15·2, 41·8)	32·3 (19·4, 45·3)	36·0 (22·8, 49·3)	0·68	0·69	0·43	0·69	0·69	0·69
Isobutyrate	Visit 2	4·12 (2·26, 5·97)	3·63 (1·59, 5·66)	5·70 (3·32, 8·08)	0·72	0·72	0·29	0·44	0·19	0·44
	Visit 3	3·62 (1·34, 5·90)	2·73 (0·20, 5·26)	6·35 (4·12, 8·58)	0·60	0·60	0·092	0·14	**0·039**	0·12
Isovalerate	Visit 2	1·55 (1·02, 2·08)	1·56 (1·04, 2·09)	1·50 (0·96, 2·04)	0·98	0·98	0·89	0·98	0·87	0·98
	Visit 3	1·83 (0·86, 2·81)	2·47 (1·51, 3·42)	2·51 (1·53, 3·49)	0·36	0·54	0·34	0·54	0·95	0·95
Propionate	Visit 2	40·6 (25·7, 55·5)	51·6 (36·9., 66·3)	33·6 (18·5, 48·7)	0·30	0·45	0·51	0·51	0·092	0·28
	Visit 3	44·8 (28·0, 61·6)	47·2 (30·9, 63·6)	66·6 (49·8, 83·4)	0·84	0·84	0·071	0·16	0·10	0·16
Valerate	Visit 2	2·48 (1·29, 3·67)	2·62 (1·44, 3·80)	2·65 (1·45, 3·86)	0.87	0.97	0.84	0.97	0.97	0.97
	Visit 3	3·32 (1·32, 5·33)	3·59 (1(64, 5·55)	5·10 (3·10, 7·10)	0.85	0.85	0.22	0.43	0.29	0.43

CG, control group (placebo supplement); Lo-EG, low-dose *B. infantis*,1·0 x 10^8 CFU; Hi-EG, high-dose *B. infantis*, 1·8 x 10^{10} CFU. Data presented are LS mean (95% Confidence Interval) from ANCOVA model adjusted for baseline value. P-value from ANCOVA adjusted for baseline value. Adjusted p-values were calculated using Benjamini-Hochberg correction.

Supplementary Table 6. Fecal biomarkers (dry feces) at visits 2 and 3, FAS population

Marker	Visit	CG	Lo-EG	Hi-EG	Lo-EG v CG Unadj p-value	Lo-EG v CG Adj p-value	Hi-EG v CG Unadj p-value	Hi-EG v CG Adj p-value	Hi-EG vs Lo-EG Unadj p-value	Hi-EG vs Lo-EG Adj p-value
**All Subjects**										
Alpha-1-Antitrypsin (mg/g)	Visit 2	2·20 (1·68, 2·72)	2·81 (2·29, 3·33)	3·14 (2·61, 3·67)	0·11	0·16	**0·014**	**0·042**	0·38	0·38
	Visit 3	4·34 (3·55, 5·13)	5·13 (4·33, 5·94)	4·16 (3·35, 4·97)	0·17	0·25	0·75	0·75	0·094	0·25
Calprotectin (mg/kg)	Visit 2	1624·5 (1260·1, 1989·0)	1433·2 (1065·8, 1800·5)	1464·3 (1079·1, 1829·5)	0·47	0·78	0·52	0·78	0·94	0·94
	Visit 3	2307·9 (1708·1, 2907·8)	2548·2 (1935·0, 3161·3)	2866·7 (2269·1, 3504·3)	0·58	0·58	0·19	0·56	0·44	0·58
sIgA (mg/kg)	Visit 2	44445·8 (34325·2, 54566·4)	50550·3 (40360·9, 60739·7)	53841·8 (43436·0, 64247·6)	0·40	0·60	0·20	0·60	0·66	0·66
	Visit 3	56355·2 (45366·4, 67344·0)	61438·7 (50220·2, 72657·2)	56756·7 (45457·9, 68055·5)	0·52	0·84	0·96	0·96	0·56	0·84
**Breastfed only Subjects**										
Alpha-1-Antitrypsin (mg/g)	Visit 2	2·147 (1·366, 2·928)	3·165 (2·393, 3·938)	3·378 (2·598, 4·159)	0·069	0·10	**0·029**	0·087	0·70	0·70
	Visit 3	4·369 (3·165, 5·572)	5·915 (4·709, 7·120)	4·279 (3·075, 5·484)	0·060	0·11	0·92	0·92	0·075	0·11
Calprotectin (mg/kg)	Visit 2	1503·3 (1100·7, 1906·0)	1322·4 (924·3, 1720·4)	1122·7 (719·6, 1525·7)	0·53	0·53	0·19	0·53	0·49	0·53
	Visit 3	2313·0 (1389·0, 3236·9)	2788·2 (1863·7, 3712·8)	2968·2 (2043·3, 3893·0)	0·47	0·71	0·32	0·71	0·79	0·79
sIgA (mg/kg)	Visit 2	53339·5 (38771·9, 67907·1)	64608·1 (50229·2, 78986·9)	60772·2 (46217·2, 75327·1)	0·28	0·71	0·48	0·71	0·71	0·71
	Visit 3	69966·2 (535972, 863353)	76733·3 (603802, 930863)	65054·7 (48700·8, 81408·5)	0·56	0·68	0·68	0·68	0·32	0·68

CG, control group (placebo supplement); Lo-EG, low-dose *B. infantis*,1·0 x 10^8 CFU; Hi-EG, high-dose *B. infantis*, 1·8 x 10^{10} CFU. Data presented LS mean (95% Confidence Interval) from ANCOVA model adjusted for baseline value. Adjusted p-values were calculated using Benjamini-Hochberg correction.

Supplementary Table 7. Descriptive statistics for cytokines at visits 2 and 3, FAS population, all subjects

Marker	Visit	CG<br>(n=75)<br><br>% out of range<br>Median<br>[Q1, Q3]	Lo-EG<br>(n=73)<br><br>% out of range<br>Median<br>[Q1, Q3]	Hi-EG<br>(n=70)<br><br>% out of range<br>Median<br>[Q1, Q3]
IFN-β	Visit 2	56·0%<br>2·87<br>[1·79, 5·41]	37·0%<br>2·10<br>[1·12, 4·09]	55·7%<br>2·02<br>[1·03, 3·42]
	Visit 3	37·3%<br>2·06<br>[1·06, 3·81]	38·4%<br>3·07<br>[1·49, 3·81]	50·0%<br>1·98<br>[0·82, 5·45]
IFN-γ	Visit 2	30·7%<br>0·53<br>[0·34, 1·34]	34·2%<br>0·42<br>[0·24, 0·78]	38·6%<br>0·40<br>[0·28, 0·60]
	Visit 3	24·0%<br>0·62<br>[0·36, 1·12]	21·9%<br>0·57<br>[0·29, 1·14]	35·7%<br>0·55<br>[0·29, 0·82]
IL-10	Visit 2	98·7%<br>0·32<br>[0·32, 0·32]	90·4%<br>0·04<br>[0·04, 0·05]	97·1%<br>0·03<br>[0·03, 0·04]
	Visit 3	89·3%<br>0·09<br>[0·05, 0·10]	83·6%<br>0·05<br>[0·03, 0·09]	85·7%<br>0·08<br>[0·03, 0·14]
IL-13	Visit 2	85·3%<br>0·55<br>[0·47, 0·68]	86·3%<br>0·54<br>[0·37, 0·93]	81·4%<br>0·59<br>[0·40, 0·78]
	Visit 3	80·0%<br>0·83<br>[0·56, 1·10]	79·5%<br>0·54<br>[0·43, 0·63]	81·4%<br>0·80<br>[0·55, 0·85]
IL-17A Gen.B	Visit 2	72·0%<br>1·17<br>[0·63, 1·78]	68·5%<br>0·94<br>[0·55, 1·43]	81·4%<br>0·83<br>[0·72, 0·89]
	Visit 3	68·0%<br>0·85<br>[0·53, 1·27]	68·5%<br>0·78<br>[0·67, 1·07]	75·7%<br>0·75<br>[0·54, 1·16]
IL-1β	Visit 2	5·3%<br>1·834<br>[0·63, 4·83]	2·7%<br>1·39<br>[0·40, 3·13]	11·4%<br>0·97<br>[0·46, 2·71]
	Visit 3	8·0%<br>1·12<br>[0·33, 4·31]	12·3%<br>0·92<br>[0·39, 2·62]	10·0%<br>0·68<br>[0·21, 2·85]
IL-6	Visit 2	86·7%<br>0·10<br>[0·06, 0·13]	82·2%<br>0·09<br>[0·07, 0·11]	77·1%<br>0·11<br>[0·06, 0·16]
	Visit 3	69·3%<br>0·12<br>[0·08, 0·18]	61·6%<br>0·09<br>[0·07, 0·16]	62·9%<br>0·08<br>[0·07, 0·13]
IL-8	Visit 2	8·0%<br>1·68<br>[0·49, 3·71]	12·3%<br>0·85<br>[0·38, 1·97]	5·7%<br>0·81<br>[0·42, 3·17]
	Visit 3	14·7%<br>1·59<br>[0·57, 5·15]	4·1%<br>0·95<br>[0·42, 2·62]	5·7%<br>1·16<br>[0·49, 3·46]
IL-21	Visit 2	56·0%<br>3·86<br>[2·86, 5·44]	64·4%<br>2·92<br>[2·46, 4·10]	57·1%<br>3·16<br>[2·30, 3·50]
	Visit 3	62·7%<br>3·82<br>[2·64, 4·62]	67·1%<br>3·09<br>[2·26, 4·05]	61·4%<br>2·72<br>[2·02, 4·25]
IL-22	Visit 2	94·7%<br>1·20<br>[0·60, 2·54]	100%	95·7%<br>0·25<br>[0·24, 0·77]
	Visit 3	88·0%<br>0·59<br>[0·42, 1·09]	94·5%<br>0·37<br>[0·24, 0·50]	97·1%<br>0·88<br>[0·37, 1·40]
IL-23	Visit 2	25·3%<br>1·91<br>[1·39, 2·74]	43·8%<br>2·13<br>[1·60, 3·38]	31·4%<br>2·06<br>[1·35, 3·66]

Marker	Visit	CG (n=75) % out of range Median [Q1, Q3]	Lo-EG (n=73) % out of range Median [Q1, Q3]	Hi-EG (n=70) % out of range Median [Q1, Q3]
	Visit 3	26·7%<br>1·87<br>[1·46, 2·69]	27·4%<br>2·34<br>[1·34, 3·78]	30·0%<br>1·68<br>[1·24, 2·56]
IL-27	Visit 2	38·7%<br>19·23<br>[15·10, 51·18]	32·9%<br>17·73<br>[12·48, 31·46]	30·0%<br>17·49<br>[12·11, 28·52]
	Visit 3	32·0%<br>18·31<br>[10·66, 31·42]	27·4%<br>14·47<br>[10·26, 23·77]	38·6%<br>12·85<br>[8·68, 21·88]
IL-31	Visit 2	98·7%<br>1·33<br>[1·33, 1·33]	98·6%<br>0·87<br>[0·87, 0·87]	95·7%<br>0·62<br>[0·53, 0·90]
	Visit 3	96·0%<br>0·48<br>[0·48, 0·48]	97·3%	100%
IL-33	Visit 2	93·3%<br>0·10<br>[0·07, 0·43]	84·9%<br>1·11<br>[0·06, 0·12]	84·3%<br>0·09<br>[0·07, 0·20]
	Visit 3	73·3%<br>0·22<br>[0·11, 0·30]	64·4%<br>0·17<br>[0·09, 0·31]	81·4%<br>0·22<br>[0·14, 0·48]
MIP-3α	Visit 2	65·3%<br>1·33<br>[1·07, 3·02]	60·3%<br>1·09<br>[0·80, 2·50]	60·0%<br>1·06<br>[0·71, 1·50]
	Visit 3	69·3%<br>1·35<br>[0·94, 1·90]	56·2%<br>1·36<br>[1·11, 1·93]	75·7%<br>0·735<br>[0·64, 0·95]
TNF-α	Visit 2	73·3%<br>0·08<br>[0·07, 0·12]	58·.9%<br>0·11<br>[0·08, 0·1.6]	67·1%<br>0·12<br>[0·11, 0·17]
	Visit 3	54·7%<br>0·14<br>[0·10, 0·19]	58·9%<br>0·14<br>[0·10, 0·20]	58·6%<br>0·13<br>[0·10, 0·15]

Units: pg/mg; CG, control group (placebo supplement); Lo-EG, low-dose *B. infantis*,1.0 x 10^8 CFU; Hi-EG, high-dose *B. infantis*, 1.8 x 10^{10} CFU.

Supplementary Table 8. ANCOVA results for cytokines with at least 50% of values above limit of detection at visits 2 and 3, FAS population, all subjects and breastfed only subjects

		**Lo-EG v CG**			**Hi-EG v CG**			**Hi-EG v Lo-EG**		
**Marker**	**Visit**	**LS mean difference (95% CI)**	**Unadjusted p-value**	**Adjusted p-value**	**LS mean difference (95% CI)**	**Unadjusted p-value**	**Adjusted p-value**	**LS mean difference (95% CI)**	**Unadjusted p-value**	**Adjusted p-value**
					**All subjects**					
IFN-γ	Visit 2	-0·067 (-3·48, 3·34)	0·97	0·97	1·87 (-1·59, 5·33)	0·29	0·43	1·94 (-1·58, 5·45)	0·28	0·43
	Visit 3	-0·11 (-0·90, 0·69)	0·79	0·79	-0·61 (-1·43, 0·20)	0·14	0·34	-0·51 (-1·33, 0·32)	0·23	0·34
IL-1β	Visit 2	-2·05 (-4·48, 0·38)	0·097	0·15	-2·83 (-5·40,-0·26)	**0·031**	0·09	-0·78 (-3·31, 1·75)	0·54	0·54
	Visit 3	-2·29 (-5·11, 0·52)	0·11	0·33	-1·69 (-4·54, 1·16)	0·24	0·37	0·60 (-2·29, 3·49)	0·68	0·68
IL-8	Visit 2	-1·52 (-4·00, 0·96)	0·23	0·52	-0·33 (-2·72, 2·06)	0·78	0·78	1·19 (-1·30, 3·68)	0·35	0·52
	Visit 3	-1·84 (-5·42, 1·74)	0·31	0·47	-0·003 (-3·49, 3·49)	>0·99	>0·99	1·84 (-1·67, 5·34)	0·30	0·47
IL-23	Visit 2	2·90 (-3·48, 9·27)	0·37	0·55	-0·56 (-6·55, 5·44)	0·85	0·85	-3·45 (-9·63, 2·72)	0·27	0·55
	Visit 3	-3·84 (-10·96, 3·29)	0·29	0·68	-1·47 (-8·43, 5·49)	0·68	0·68	2·36 (-4·23,8·96)	0·48	0·68
IL-27	Visit 2	-10·87 (-29·69, 7·94)	0·25	0·42	-10·39 (-29·31,8·53)	0·28	0·42	0·48 (-18·24,19·20)	0·96	0·96
	Visit 3	-8·71 (-30·46, 13·05)	0·43	0·55	-15·53 (-38·43, 7·36)	0·18	0·54	-6·82 (-29·50, 15·80)	0·55	0·55
					**Breastfed only subjects**					
IFN-γ	Visit 2	-0·24 (-5·52, 5·03)	0·93	0·93	3·00 (-2·34, 8·34)	0·27	0·40	3·24 (-2·27, 8·75)	0·25	0·40
	Visit 3	-0·82 (-1·75, 0·10)	0·080	0·12	-0·87 (-1·80, 0·07)	0·070	0·12	-0·04 (-1·01, 0·92)	0·93	0·93
IL-1β	Visit 2	-5·77 (-9·19, -2·35)	**0·0011**	**0·0034**	-4·73 (-8·28, -1·18)	**0·0095**	**0·014**	1·04 (-2·39, 4·47)	0·55	0·55
	Visit 3	-3·17 (-5·90, -0·45)	**0·023**	**0·035**	-3·97 (-6·71, -1·23)	**0·0050**	**0·015**	-0·80 (-3·50, 1·90)	0·56	0·56
IL-8	Visit 2	-3·39 (-7·10, 0·32)	0·073	0·22	-1·38 (-4·92, 2·16)	0·44	0·44	2·01 (-1·61, 5·63)	0·27	0·41
	Visit 3	-5·47 (-9·53, -1·41)	**0·0087**	**0·026**	-4·09 (-7·99, -0·18)	**0·041**	0·061	1·39 (-2·48, 5·26)	0·48	0·48
IL-23	Visit 2	-2·23 (-5·47, 1·01)	0·17	0·32	-2·00 (-5·18, 1·19)	0·21	0·32	0·23 (-2·80, 3·26)	0·88	0·88
	Visit 3	-0·55 (-10·85, 9·75)	0·92	0·92	3·87 (-6·62, 4·35)	0·46	0·69	4·41 (-5·06, 3·89)	0·35	0·69
IL-27	Visit 2	-8·01 (-34·05, 18·03)	0·54	0·78	-11·73 (-38·47, 15·01)	0·38	0·78	-3·72 (-30·15, 22·71)	0·78	0·78
	Visit 3	-11·64 (-43·64, 20·37)	0·47	0·71	-17·07 (-53·20, 19·06)	0·35	0·71	-5·43 (-41·46, 30·60)	0·76	0·76

CG, control group (placebo supplement); Lo-EG, low-dose *B. infantis*,1·0 x 10^8 CFU; Hi-EG, high-dose *B. infantis*, 1·8 x 10^{10} CFU. ANCOVA includes adjustment for baseline value. Adjusted p-values were calculated using Benjamini-Hochberg correction.

Supplementary Table 9. Statistical summary of group alpha diversity differences for gene, MGS, and genus-level richness.

Visit	Taxonomical level	Diversity measure	CG Median [Q1, Q3]	Lo-EG Median [Q1, Q3]	Hi-EG Median [Q1, Q3]	Kruskal-Wallis p-value	Dunn's p-value CG v Lo-EG	Dunn's p-value CG v Hi-EG	Dunn's p-value Lo-EG v Hi-EG
Visit 1	Gene	Richness	95871 [78841, 120174]	101899 [76456, 123403]	101519 [74321, 126164]	0·843	n/a	n/a	n/a
		Shannon	10.2 [10.0, 10.6]	10.2 [9.7, 10.5]	10.2 [9.7, 10.5]	0·687	n/a	n/a	n/a
	MGS	Richness	41 [33, 51]	42 [33, 55]	43 [30, 54]	0·981	n/a	n/a	n/a
		Shannon	1.9 [1.6, 2.2]	1.9 [1.5, 2.2]	1.9 [1.5, 2.2]	0·508	n/a	n/a	n/a
		Faith's PD	19.5 [15.3, 23.3]	19.6 [15.4, 23.1]	19.6 [15, 25]	0·894	n/a	n/a	n/a
	Genus	Richness	17 [14, 21]	17 [14, 21]	18 [14, 23]	0·754	n/a	n/a	n/a
		Shannon	1.5 [1.2, 1.7]	1.3 [1, 1.7]	1.4 [0.9, 1.7]	0·401	n/a	n/a	n/a
Visit 2	Gene	Richness	109237 [90153, 139724]	103989 [78010, 130565]	96739 [71156, 128602]	0·234	n/a	n/a	n/a
		Shannon	10.2 [9.9, 10.5]	10.1 [9.6, 10.4]	10 [9.5, 10.4]	0·074	n/a	n/a	n/a
	MGS	Richness	51 [40, 61]	47 [37, 61]	47 [33, 58]	0·434	n/a	n/a	n/a
		Shannon	1.9 [1.6, 2.2]	1.8 [1.4, 2.2]	1.7 [1.3, 2.1]	0·400	n/a	n/a	n/a
		Faith's PD	23.6 [18.9, 27.1]	22.5 [17.3, 28.7]	23.6 [17.8, 26.6]	0·984	n/a	n/a	n/a
	Genus	Richness	22 [17, 25]	20 [16, 26]	21 [14, 25]	0·524	n/a	n/a	n/a
		Shannon	1.3 [1.0, 1.7]	1.1 [0.6, 1.5]	1.0 [0.7, 1.6]	**0·00521**	**0·0045**	**0·00602**	0·895
Visit 3	Gene	Richness	110780 [88167, 132630]	109606 [92956, 138893]	119724 [91470, 138967]	0·510	n/a	n/a	n/a
		Shannon	10 [9.7, 10.4]	10.2 [9.8, 10.4]	10.1 [9.7, 10.5]	0·310	n/a	n/a	n/a
	MGS	Richness	51 [38, 64]	53 [41, 69]	56 [43, 70]	0·415	n/a	n/a	n/a
		Shannon	1.7 [1.4, 2.1]	1.9 [1.4, 2.2]	1.8 [1.4, 2.3]	0·468	n/a	n/a	n/a
		Faith's PD	24.7 [18.9, 29.3]	25.7 [19.5, 30.5]	24.3 [20.3, 31.1]	0·383	n/a	n/a	n/a
	Genus	Richness	24 [17, 28]	23 [17, 28]	23 [18, 29]	0·522	n/a	n/a	n/a
		Shannon	1.2 [0.9, 1.6]	1.2 [0.7, 1.5]	1.1 [0.8, 1.6]	0·394	n/a	n/a	n/a
Visit 4	Gene	Richness	129254 [107885, 163289]	128683 [102894, 157331]	123937 [87304, 156408]	0·559	n/a	n/a	n/a
		Shannon	10.3 [10, 10.6]	10.2 [9.9, 10.6]	10.1 [9.8, 10.6]	0·419	n/a	n/a	n/a
	MGS	Richness	57 [45, 78]	60 [45, 73]	56 [42, 71]	0·577	n/a	n/a	n/a
		Shannon	2.0 [1.6, 2.3]	1.9 [1.6, 2.4]	1.9 [1.4, 2.3]	0·832	n/a	n/a	n/a
		Faith's PD	27.2 [21.1, 33.6]	28.6 [21.7, 33.1]	25.2 [20.4, 31.1]	0·270	n/a	n/a	n/a
	Genus	Richness	25 [19, 32]	28 [20, 32]	23 [19, 30]	0·312	n/a	n/a	n/a
		Shannon	1.4 [1.1, 1.7]	1.3 [0.9, 1.7]	1.3 [0.9, 1.7]	0·470	n/a	n/a	n/a

CG, control group (placebo supplement); Lo-EG, low-dose *B. infantis*,1·0 x 10^8 CFU; Hi-EG, high-dose *B. infantis*, 1·8 x 10^{10} CFU. Shannon diversity (Shannon), and MGS-level Faith's Phylogenetic distance (Faith's PD). Pairwise comparisons using Dunn's test were applied for measures significantly different in the Kruskal-Wallis test.

Supplementary Table 10. PERMANOVA R^2 and p-values based on 1,000 permutations for overall and pairwise comparisons of Bray-Curtis dissimilarity and weighted UniFrac distance between groups at each visit.

		Bray-Curtis		Weighted UniFrac	
**Visit**	**Comparisons**	**R^2**	**p-value**	**R^2**	**p-value**
Visit 1	All	0·87%	0·560	0·92%	0·398
	CG vs Lo-EG	0·58%	0·654	1·22%	0·093
	CG vs Hi-EG	0·73%	0·405	0·78%	0·297
	Lo-EG vs Hi-EG	0·66%	0·536	0·05%	0·988
Visit 2	All	**4·93%**	**0·001**	**3·1%**	**0·012**
	CG vs Lo-EG	**5·13%**	**0·001**	**2·94%**	**0·007**
	CG vs Hi-EG	**5·00%**	**0·001**	**3·52%**	**0·012**
	Lo-EG vs Hi-EG	0·48%	0·713	0·18%	0·906
Visit 3	All	**2·83%**	**0·001**	1·22%	0·216
	CG vs Lo-EG	**2·71%**	**0·001**	1·26%	0·104
	CG vs Hi-EG	**2·72%**	**0·002**	1·18%	0·144
	Lo-EG vs Hi-EG	0·76%	0·341	0·19%	0·871
Visit 4	All	**1·92%**	**0·005**	0·87%	0·427
	CG vs Lo-EG	**1·81%**	**0·003**	0·82%	0·273
	CG vs Hi-EG	**1·95%**	**0·006**	0·82%	0·269
	Lo-EG vs Hi-EG	0·46%	0·768	0·29%	0·833

CG, control group (placebo supplement); Lo-EG, low-dose *B. infantis*,1·0 x 10^8 CFU; Hi-EG, high-dose *B. infantis*, 1·8 x 10^{10} CFU.

Supplementary Table 11. Significantly different taxa between groups per visit. P-values were adjusted using FDR applied to each taxonomical rank separately. No taxa were significantly different at Visit 1.

			**Median abundance (%) [IQR1; IQR3]**				**Dunn's test adjusted p-value**		
**Visit**	**Rank**	**Taxon**	**CG**	**Lo-EG**	**Hi-EG**	**Kruskal-Wallis adjusted p-value**	**CG v Lo-EG**	**CG v Hi-EG**	**Lo-EG v Hi-EG**
Visit 2	MGS	*B. infantis*	0 [0; 0.005]	22.7 [4.3; 47.8]	21.6 [3.8; 51.5]	$3{\cdot}4*10^{-12}$	$1{\cdot}1*10^{-11}$	$1{\cdot}1*10^{-11}$	0·973
	genus	*Bifidobacterium*	41.5 [12.5; 70.0]	69.3 [30.7; 83.8]	66.4 [34.8; 82.7	0·086	0·0061	0·0040	0·709
	genus	*Klebsiella*	3.85 [0.95; 15.4]	0.77 [0.14; 6.2]	1.05 [0.098; 10.4]	0·086	0·0029	0·019	0·451
	genus	*Mediterraneibacter*	0.0066 [0; 0.60]	0 [0; 0.18]	0.0023 [0; 0.017]	0·086	0·243	0·0027	0·046
	-	Infant-type Bifidobacteria	31.0 [1.3; 67.4]	59.0 [23.9; 83.0]	59.9 [26.1; 82.3]	0·00038	0·00080	0·00027	0·616
Visit 3	MGS	*B. infantis*	0 [0; 9.8]	21.8 [1.0; 53.7]	11.7 [1.7; 45.8]	$3{\cdot}1*10^{-7}$	$1{\cdot}7*10^{-8}$	$2{\cdot}9*10^{-7}$	0·574
	-	Infant-type Bifidobacteria	41.9 [8.8; 67.9]	61.8 [19.6; 81.0]	61.4 [25.0; 79.7]]	0·038	0·013	0·013	0·949
Visit 4	MGS	*B. infantis*	0 [0; 1.5]	14.6 [0.02; 44.1]	11.9 [0.05; 51.4]	0·00020	$8{\cdot}8*10^{-6}$	$8{\cdot}8*10^{-6}$	0·982
	MGS	*Lachnospiraceae sp.*	0.0 [0.0; 0.0]	0.0 [0.0; 0.0]	0.0 [0.0; 0.0]	0·0082	0·00082	0·00014	0·512
	-	Infant-type Bifidobacteria	48.2 [12.9; 63.6]	52.2 [22.8; 76.5]	60.8 [28.8; 80.7]	0·0832	0·038	0·034	0·743

CG, control group (placebo supplement); Lo-EG, low-dose *B. infantis*, $1{\cdot}0 \times 10^{8}$ CFU; Hi-EG, high-dose *B. infantis*, $1{\cdot}8 \times 10^{10}$ CFU. IQR, Inter Quartile Range. P-values were adjusted using FDR applied to each taxonomical rank separately. No taxa were significantly different at Visit 1.

Supplementary Table 12. Prevalence of pathogenic species identified by detection of specific virulence factors among groups and visits.

Pathogen	Visit	CG	Lo-EG	Hi-EG
Campylobacter spp.	V1	0	1 (1·4%)	0
	V2	0	1 (1·4%)	2 (2·9%)
	V3	4 (5·5%)	0	0
	V4	0	1 (1·4%)	0
Clostridioides difficile	V1	2 (2·7%)	0	1 (1·4%)
	V2	10 (13%)	4 (5·5%)	4 (5·8%)
	V3	14 (19%)	7 (9.6%)	7 (10%)
	V4	17 (23%)	14 (19%)	10 (14%)
Clostridium perfringens	V1	1 (1·4%)	0	0
	V2	0	1 (1·4%)	0
	V3	0	0	0
	V4	0	0	0
Escherichia coli	V1	1 (1·4%)	0	0
	V2	1 (1·3%)	1 (1·4%)	0
	V3	0	0	3 (4·3%)
	V4	4 (5·3%)	2 (2·7%)	3 (4·3%)

CG, control group (placebo supplement); Lo-EG, low-dose *B. infantis*,$1{\cdot}0 \times 10^{8}$ CFU; Hi-EG, high-dose *B. infantis,* $1{\cdot}8 \times 10^{10}$ CFU. Numbers in brackets refer to the percentage of all infants in the group at the visit.

Supplementary Table 13. Chi-squared test p-values for overall and pairwise differences in FCT profiles between samples stratified according to *B. infantis* strain colonization at V4.

Visit	p-value (overall)	p-value (pairwise; LMG11588 vs No B. infantis)	p-value (pairwise; Other B. infantis vs No B. infantis)	p-value (pairwise; Other B. infantis vs LMG11588)
Visit 1	0·009	0·020	$9·47 *10^{-4}$	0·246
Visit 2	$2·23 *10^{-4}$	0·001	$7·11 *10^{-5}$	0·381
Visit 3	$2·61 *10^{-4}$	$8·48 *10^{-4}$	$2·27 *10^{-4}$	0·334
Visit 4	$6·12 *10^{-7}$	$5·6 *10^{-5}$	$2·34 *10^{-5}$	0·101
